# TNF superfamily control of tissue remodeling and fibrosis

**DOI:** 10.3389/fimmu.2023.1219907

**Published:** 2023-07-03

**Authors:** Hope Steele, Jason Cheng, Ashley Willicut, Garrison Dell, Joey Breckenridge, Erica Culberson, Andrew Ghastine, Virginie Tardif, Rana Herro

**Affiliations:** ^1^ Division of Immunobiology, Cincinnati Children’s Hospital Medical Center, Cincinnati, OH, United States; ^2^ University of Cincinnati, Cincinnati, OH, United States; ^3^ University of Cincinnati College of Medicine, Cincinnati, OH, United States; ^4^ Normandy University, UniRouen, Institut National de la Santé et de la Recherche Médicale (INSERM), UMR1096 (EnVI Laboratory), Rouen, France; ^5^ Department of Pediatrics, University of Cincinnati, Cincinnati, OH, United States

**Keywords:** TNF superfamily, TNFSF, fibrosis, remodeling, mucosa

## Abstract

Fibrosis is the result of extracellular matrix protein deposition and remains a leading cause of death in USA. Despite major advances in recent years, there remains an unmet need to develop therapeutic options that can effectively degrade or reverse fibrosis. The tumor necrosis super family (TNFSF) members, previously studied for their roles in inflammation and cell death, now represent attractive therapeutic targets for fibrotic diseases. In this review, we will summarize select TNFSF and their involvement in fibrosis of the lungs, the heart, the skin, the gastrointestinal tract, the kidney, and the liver. We will emphasize their direct activity on epithelial cells, fibroblasts, and smooth muscle cells. We will further report on major clinical trials targeting these ligands. Whether in isolation or in combination with other anti-TNFSF member or treatment, targeting this superfamily remains key to improve efficacy and selectivity of currently available therapies for fibrosis.

## 1 Introduction

**Figure 1:**
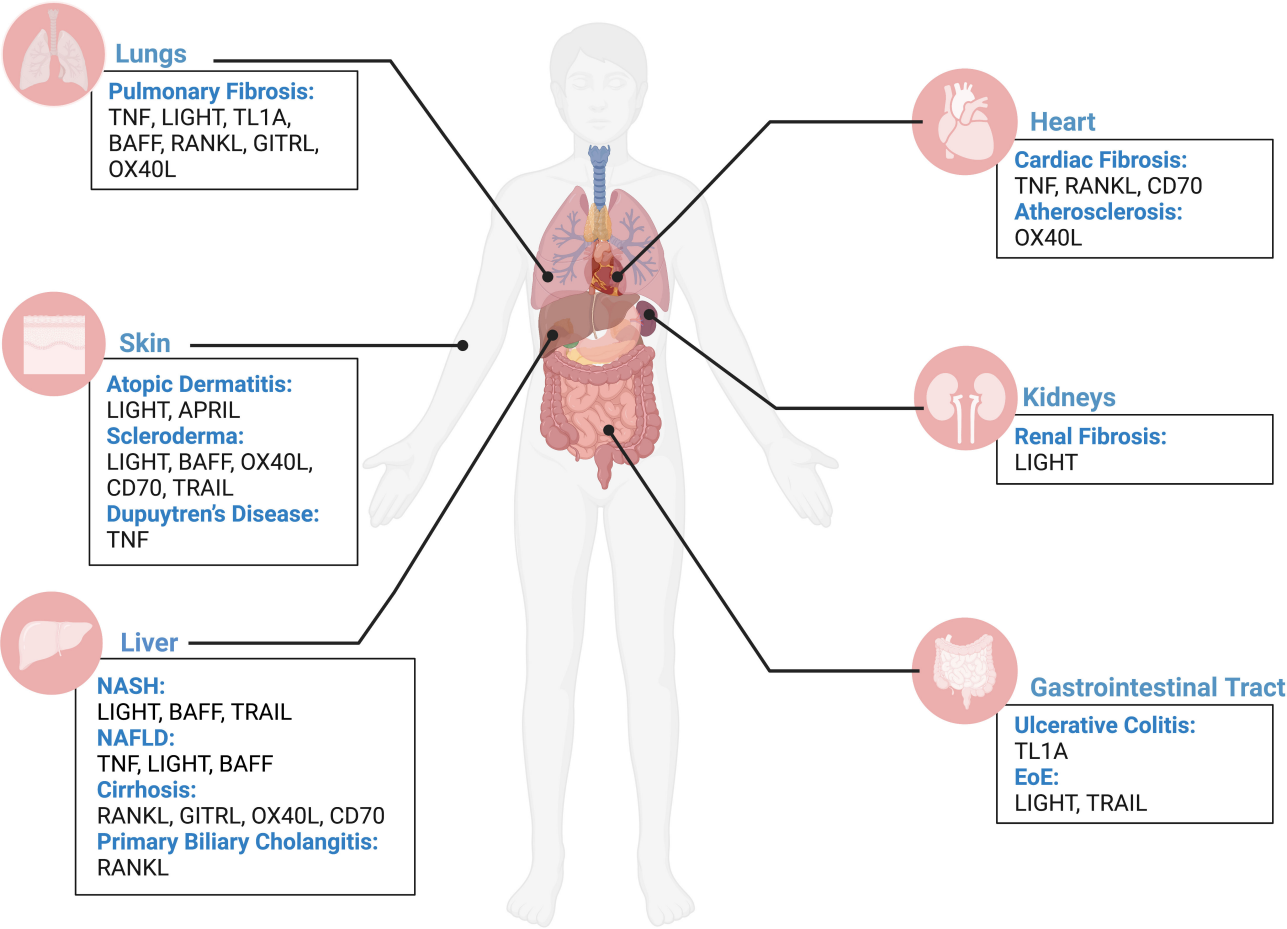
TNFSF contribution to fibrosis across human organs. Overview of contribution of TNFSF members in driving fibrotic disease in the lungs, heart, skin, kidneys, liver, and gastrointestinal tract based on current literature. Figure was created with BioRender.com.

**Figure 2:**
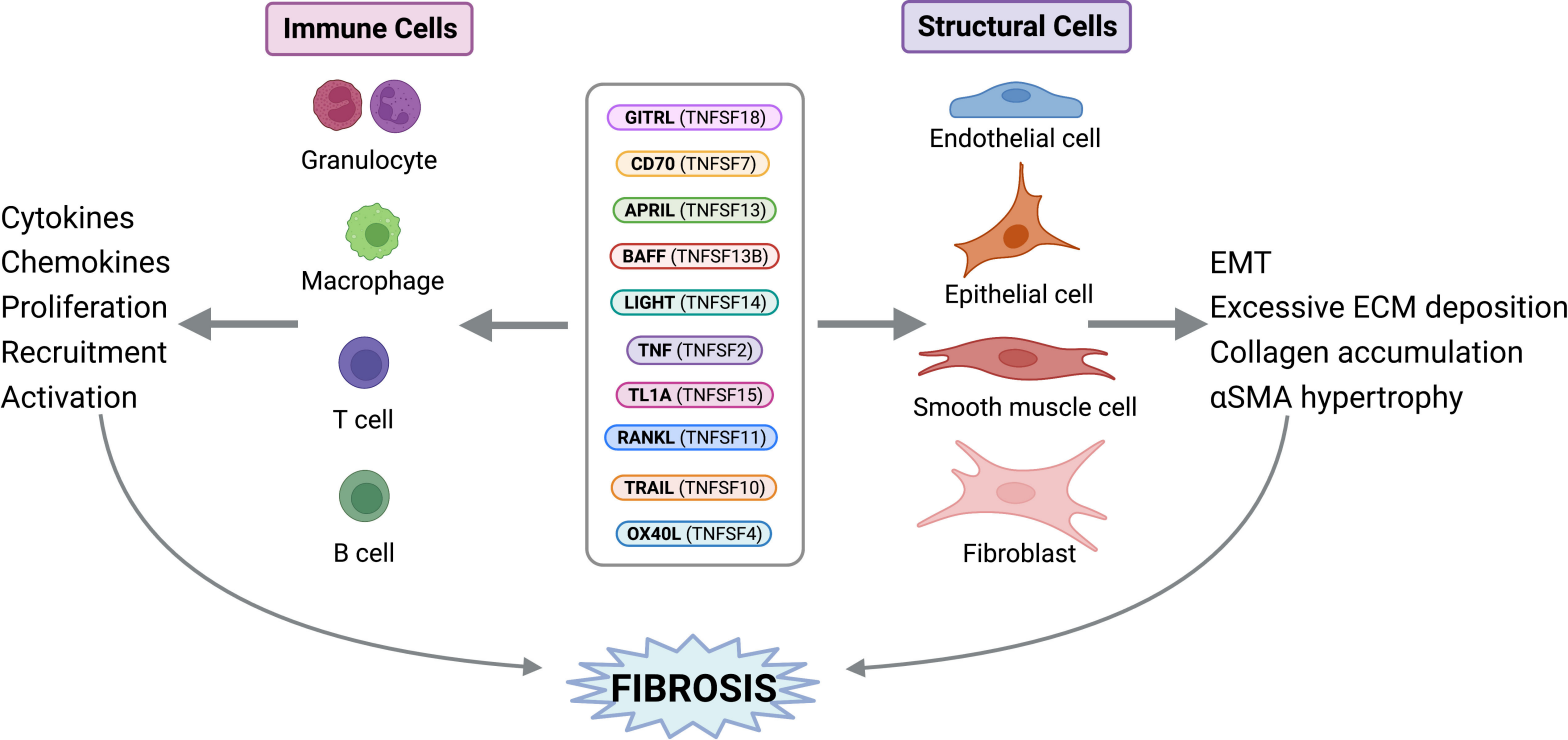
TNF Superfamily Member Activity on Structural and Immune Cells. TNFSF members can exert direct activities on structural and immune cells to perpetuate fibrosis. Figure was created with BioRender.com.

Fibrosis is defined as the uncontrolled build-up of scar tissue. It is the end result of many inflammatory diseases and is responsible for 45% of death in USA (1). Fibrosis can affect multiple organs and, irrelevant of the initiating disorder, it can ultimately lead to organ failure and death. Fibrosis originates after epithelial (or endothelial) injury, driving early alarmin expression, followed by inflammation that involves the production of the central fibrogenic cytokine TGFβ (transforming growth factor-beta). TGFβ allows for the differentiation of fibroblasts into pathogenic myofibroblasts. Myofibroblasts are responsible for collagen and extracellular matrix proteins deposition, in addition to smooth muscle hypertrophy. While TGFβ is central for fibrosis pathogenesis, its’ targeting leads to severe side effects due to lymphoproliferative symptoms. Therefore, there is an urgent need to identify novel fibrotic mediators that could be therapeutic targets for the myriad of diseases presenting with fibrosis. Historically, tumor necrosis factor superfamily (TNFSF) ligands have been identified as proinflammatory mediators; however, our group has pioneered work implicating the TNF superfamily members in fibrosis (2–5). Currently, the TNF superfamily is known to consist of 19 ligands and 29 receptors. Current literature demonstrates growing evidence that the TNFSF members covered in this review (**Table 1**) play a role in fibrosis, including their upregulation in various fibrotic disorders; however, this does not rule out the potential contributions of other TNFSF members, which future research should address. In this review, we will summarize select TNFSF (**Table 1**) and their involvement in fibrosis of the lungs (interstitial lung disease, pulmonary fibrosis, idiopathic pulmonary fibrosis, acute respiratory distress syndrome, cystic fibrosis), the heart (atherosclerosis, myocarditis, ischemic myocardial infarction, non-ischemic hypertrophic cardiomyopathy), the skin (scleroderma, atopic dermatitis, atopic eczema, Dupuytren’s disease), the gastrointestinal tract (eosinophilic esophagitis, ulcerative colitis, Crohn’s disease), the kidney (acute kidney injury, chronic kidney disease), and the liver (non-alcoholic steatohepatitis, non-alcoholic fatty liver disease, primary biliary cholangitis) (**Figure 1**). We will emphasize their direct activity on stromal cells that perpetuate fibrosis, namely epithelial cells, fibroblasts, and smooth muscle cells (**Figure 2**). We will further report on major active clinical trials targeting these ligands. Whether targeting each TNFSF ligand in isolation or in combination with another TNFSF member or treatment, targeting this superfamily remains key to improve efficacy and selectivity of currently available therapies for fibrosis.

**Table 1:** TNF superfamily members and receptors implicated in fibrosis.

Ligand	TNFSF Nomenclature	Receptor	TNFRSF Nomenclature
**TNF** (TNFα)	TNFSF2	**TNFR1** (CD120a, TNFAR, p55)	TNFRSF1A
		**TNFR2** (CD120b, TNFBR, p75)	TNFRSF1B
**LIGHT** (CD258, HVEM-L)	TNFSF14	**HVEM** (CD270, ATAR, TR2)	TNFRSF14
		**LTβR** (CD18, TNFCR)	TNFRSF3
		**DcR3** (TR6, M68)	TNFRSF6B
**TL1A** (VEGI, TL1)	TNFSF15	**DR3** (Apo-3, TRAMP, LARD, WS-1)	TNFRSF25
		**DcR3** (TR6, M68)	TNFRSF6B
**APRIL** (CD256, TALL-2)	TNFSF13	**TACI** (CD267, IGAD2)	TNFRSF13B
		**BCMA** (CD269)	TNFRSF13A
**BAFF** (CD257, BLyS, TALL-2)	TNFSF13B	**TACI** (CD267, IGAD2)	TNFRSF13B
		**BCMA** (CD269, BCM)	TNFRSF13A
		**BAFFR** (CD268)	TNFRSF13C
**RANKL** (CD254, TRANCE, OPGL, ODF)	TNFSF11	**RANK** (CD265, ODFR, FEO)	TNFRSF11A
		**OPG** (OCIF, TR1)	TNFRSF11B
**GITRL** (TL6, AITRL)	TNFSF18	**GITR** (CD357, AITR)	TNFRSF18
**OX40L** (CD252, CD134L, Gp34, TXGP1)	TNFSF4	**OX40** (CD134, ACT35, TXGP1L)	TNFRSF4
**CD70** (CD27L)	TNFSF7	**CD27** (S152, Tp55)	TNFRSF7
**TRAIL** (CD253, APO-2L, TL2)	TNFSF10	**DR4** (CD261, TRAILR1, Apo-2)	TNFRSF10A
		**DR5** (CD262, TRAILR2, KILLER)	TNFRSF10B
		**DcR1** (CD263, TRAILR3, LIT, TRID)	TNFRSF10C
		**DcR2** (CD264, TRAILR4, TRUNDD)	TNFRSF10D

TNFSF members and their corresponding receptors discussed in this review article. Common ligand and corresponding receptor names are listed in bold. Alternative ligand and receptor names are listed in parenthesis.

## 2 TNF : TNFR1/TNFR2

Tumor necrosis factor (TNF), also referred to as TNFα or TNFSF2, is a pleiotropic cytokine known for its early and potent role in inflammation (6, 7). TNF is first membrane bound (mTNF) on the cell surface and can then be cleaved by TNF-converting enzyme (TACE), a member of the disintegrin and metalloproteinase family, into its soluble form (sTNF) (8). TNF is produced primarily by monocytes and macrophages; however, it can also be expressed by other cell types such as T cells, B cells, NK cells, mast cells, neutrophils, and fibroblasts (9). TNF has two transmembrane receptors: TNF receptor I (TNFR1, also known as TNFRα, p55, or CD120a) and TNF receptor II (TNFR2, also known as TNFRβ or p75) (10). TNFR1 is ubiquitously expressed across human tissues, while TNFR2 is limited to mainly hematopoietic and endothelial cells (11). TNFR1 can be stimulated by either mTNF or sTNF, while TNFR2 is preferentially activated by mTNF (12–14). TNFR1 and TNFR2 share similar extracellular structures but, their intracellular structures differ. The cytoplasmic tail of TNFR1 contains a death domain (DD), which can recruit TNFR1-associated DD (TRADD), while TNFR2 instead recruits TNFR-associated factor (TRAF) 1 and 2 proteins (15, 16). Due to their intracellular domains, both TNFR1 and TNFR2 may signal to induce NF-κB activation and cell survival responses, whereas TNFR1 also has potential to induce cell death response.

**Figure 3:**
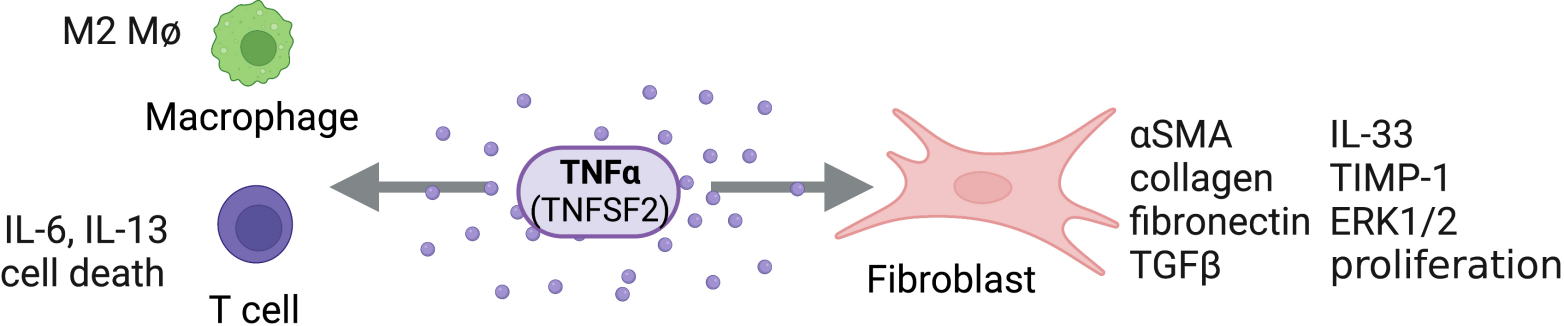
TNFα Activity on Structural and Immune Cells. TNFα (TNFSF2) can act directly on structural cells (fibroblasts) and immune cells (macrophages, T cells) to drive pro-fibrotic and anti-fibrotic effects. Figure was created with BioRender.com.

Food and Drug Administration (FDA)-approved TNF antagonists (infliximab, etanercept, adalimumab, certolizumab and golimumab) have shown to be extremely efficacious in the treatment of inflammatory diseases presenting with end-stage fibrosis, such as rheumatoid arthritis, spondylarthropathies, Crohn’s disease, and ulcerative colitis. Historically, the role of TNF in fibrosis was considered controversial, with many perceiving TNF as antifibrotic; however, current research suggests that targeting TNF, either directly or by its receptors, could present a promising approach for treating fibrotic diseases (**Figure 3**).

Systemic sclerosis (SSc) is an idiopathic autoimmune disease that presents with fibrosis mainly in the skin but also in visceral organs including the lungs and heart. Elevated serum concentration of soluble TNFR1 correlates with SSc disease severity (17, 18) and soluble TNFR1 levels are increased in the bleomycin-induced murine model of SSc (19). TACE processes pro-TNF and its receptors including soluble TNFR1. Blocking TACE, using tumor necrosis factor-alpha processing inhibitor-1 (TAPI-1), reduced skin thickness, the number of myofibroblasts, and mRNA expression of Col1a1, TGFβ, and αSMA (alpha smooth muscle actin). TACE remains a potential therapeutic target in SSc patients, though further studies are needed to understand the potential impact of TAPI-1 treatment in humans. Additional work by Hügle et al. showed that TNFR1 and TNFR2 were up regulated on dermal T cells from patients with diffuse cutaneous SSc and that TNFR2 expression correlated with skin thickening (20). Further, TNF-co-stimulation after CD3/CD28 stimulation of these SSc patient T cells resulted in elevated type 1 collagen expression by fibroblasts and an increased secretion of profibrotic cytokines by the T cells, albeit a decreased production of IL-10, indicating that SSc patient T cells may reinforce fibrosis while lacking the ability to resolve inflammation. Recent work has also shown that sTNFR1 is increased in the fibroblast supernatant from IPF patients, and this pathway can likely induce T cell death, further contributing to disease by permitting fibroblast/myofibroblast survival (21).

In a bleomycin-induced mouse model of pulmonary fibrosis, early work showed elevated TNF upon bleomycin instillation and blocking TNF prevented fibrotic disease progression, including collagen deposition (22). In later work also using the bleomycin model of pulmonary fibrosis, TNF^-/-^ and TNF^tm/tm^ (mice only expressing mTNF) mice were protected from pulmonary fibrosis as compared to WT controls (23). When recombinant TNF was added to the TNF^tm/tm^, however, collagen overexpression and fibrotic lesions were observed. This evidence, along with observations that sTNF appeared to be necessary for appropriate lymphocyte recruitment and TGF-β1 expression, led Oikonomou et al. to conclude that sTNF, as opposed to mTNF, mediates the transition from pulmonary inflammation to fibrosis. While this work focused on deletion of TNF, contrasting work by Redente et al. administered TNF as a therapeutic into the lungs of WT mice with pre-established pulmonary fibrosis driven by bleomycin (24). After pulmonary delivery of TNF, there was a decrease in fibrotic burden and number of profibrotic alternatively programmed macrophages. Furthermore, conditional macrophage depletion phenocopied the resolution seen in the mice receiving the therapeutic TNF delivery. TNF may resolve established pulmonary fibrosis by reducing numbers and/or programming status of profibrotic macrophages, however, the exact mechanism remains unclear and further work must be done to explain the apparent discrepancy that TNF is driving pulmonary fibrosis on one hand, while helping to resolve fibrosis on the other.

More recent work by Li et al. showed that impaired TNF/TNFR2 signaling enhances Th2 and Th17 polarization and aggravates allergic airway inflammation (25). Inhibiting TNFR2 via antibody treatment led to the increased expression of Th2 and Th17 inflammatory cytokines both in serum and in bronchoalveolar lavage fluid (BALF). Flow cytometry data further showed an inhibition of Th1 and CD4+CD25+ T-cell differentiation with a promotion of Th2 and Th17 polarization *in vivo*, which was subsequently replicated *in vitro*. It may be that impaired TNFR2 signaling can help facilitate traditionally fibrotic Th2/Th17 polarization, however, further studies are needed. In BALF from fibrotic and non-fibrotic hypersensitivity pneumonitis (HP) patients, transmembrane TNFR2 was elevated in fibrotic compared to non-fibrotic samples (26). In contrast, soluble TNFR2 and sTNF were both upregulated in non-fibrotic HP patients. Thus, TNFR2 signaling appears to be key for mediating fibrotic development; however, additional work needs to be done to understand whether this is a global or rather a disease-specific trend.

In human dermal fibroblasts, Goldberg et al. found that the addition of TNF *in vitro* suppresses αSMA expression (27). Similarly, while the addition of TGFβ1 increased αSMA, adding both TGFβ1 and TNF suppressed αSMA to below the baseline. Furthermore, TNF was found to suppress TGFβ1- induced myofibroblast genes at the mRNA level including Col1a1, fibronectin and αSMA. TNF-mediated inflammation may prevent TGFβ1- driven normal wound healing and fibrosis development. In contrast, in normal fibroblasts from the palm of patients with Dupuytren’s disease (DD), a localized fibrotic condition of the hand, TNF treatment drove their conversion to myofibroblasts via activation of Wnt signaling (28). Similarly, TNF inhibition by neutralizing antibodies resulted in reversal of myofibroblast phenotype, suggesting that TNF could be a promising therapeutic target for DD. In continuation of this work, Izadi et al. demonstrate that TNF signals through TNFR2 on stromal cells to initiate low-level IL-33 production (29). In turn, they argue that IL-33 can then signal via ST2 (suppression of tumorigenicity 2 aka IL1RL1) on local immune cells to further drive TNF expression. Future studies may explore inhibition of TNFR2 in combination with IL-33 inhibition, which may represent a more efficacious approach than TNFR2 inhibition alone to prevent further disease development in DD.

Stimulation of intestinal myofibroblasts with TNF increased proliferation and collagen accumulation by acting primarily via TNFR2 (30). Theiss et al. found that TNF also induced expression of tissue inhibitor of metalloproteinase-1 (TIMP-1) and activation of ERK1/2. Thus, limiting TNFR2 action may have potential for the treatment of intestinal inflammation or Crohn’s disease. In a model of liver fibrosis induced by carbon tetrachloride (CCl4) injections, deletion of TNFR1, but not TNFR2, inhibits liver fibrosis (31). TNFR1^-/-^ mice exhibited reduced procollagen and TGFβ synthesis as well as reduced levels of IL-6 mRNA as compared to WT and TNFR2^-/-^ mice, indicating that TNFR1 may play a key role in liver fibrosis formation. In a study of liver cirrhosis induced by thioacetamide in rats, Abdul-Hamid et al. tested the antifibrogenic effect of etanercept, one of the five FDA-approved TNF antagonists (32). They observed that etanercept decreased TNFR1 expression and accumulation of both collagen and hemosiderin, concluding that etanercept may attenuate fibrosis progression but also may be a therapeutic approach for hepatic iron overload-associated disorders. Finally, in a high-fat diet (HFD) mouse model of NAFLD, Wandrer et al. tested a novel antibody that selectively inhibits TNFR1, but not TNFR2 (33). Inhibition of TNFR1 markedly reduced liver steatosis, apoptotic liver injury, NAFLD activity and liver fibrosis as compared to control mice. Taken together, these studies emphasize the profibrotic role of TNFR1 in the liver, regardless of the model, and indicate that selective TNFR1 inhibition could be a promising therapeutic approach for treatment of liver fibrosis.

Cardiac inflammation, together with edema and fibrosis, are common features of many cardiovascular diseases (CVD), including ischemic myocardial infarction (MI), non-ischemic hypertrophic cardiomyopathy (HCM) and myocarditis. However, the type of fibrosis leading to heart failure (HF) will differ depending on the biological origin of HF. Upon mTNF cleavage by the TNF-alpha-converting enzyme (TACE) (34) or ADAM17 (a disintegrin and metalloproteinase 17) (35), the soluble (sTNF) form of mTNF can be released with higher affinity for TNFR1 and indirectly balance TNFR2 signaling, as shown in the control of atherosclerosis. Conversely, ADAM17 deficiency favors overactivation of TNFR2 and atherosclerosis progression in low-density lipoprotein receptor (*Ldlr*)-deficient mice (35), suggesting that each receptor has distinct modes of signaling and cellular functions. Moreover, it has also been reported that the lymphotoxin- α homotrimer (LTα3) signals through TNFR1 (36), and has unique roles in initiation and exacerbation of some inflammatory diseases. In a mouse model of HCM induced by infusion of angiotensin II (Ang II), Ang II induced the synthesis of monocyte chemoattractant protein-1 (MCP-1), which mediates the cardiac infiltration of CD34^+^CD45^+^ monocytic cells that can differentiate into collagen-producing fibroblasts that are responsible for the Ang-II-induced development of non-adaptive cardiac fibrosis. In response to continuous Ang-II, TNFR1-KO mice, but not TNFR2-KO, have shown reduced cardiac collagen deposition and CD34^+^CD45^+^ monocytic infiltration, while double receptor knockout mice were protected from cardiac fibrosis after 1 week of Ang-II infusion (37). In the same model, authors show that absence of TNFR1 signaling is essential for decreasing the amount of cardiac pro-fibrotic M2-like cells (day7 post-Ang-II), however, this loss of signal does not affect early pro-inflammatory M1-like infiltration (day 1 post-AngII). Reconstitution with WT-bone marrow into TNFR1-KO mice abrogated the protective loss of TNFR1 signaling effect by restoring cardiac M2 infiltration, upregulation of proinflammatory/profibrotic genes and development of fibrosis. Indeed, an *in vitro* mouse monocyte-to-fibroblast differentiation assay demonstrated an essential role of TNFR1 signaling in the sequential progression of monocyte activation and fibroblast formation (38). Moreover, in a model of cardiac pressure-overload induced by transaortic constriction (TAC model), TNFR2 activates the AKT pathway and inhibits the NF-κB pathway via mTNF-induced signaling, which alleviates mechanical stress and mediates cardiac hypertrophy rather than directly inducing cardiac fibrosis (39, 40).

Although ischemic, and non-ischemic cardiomyopathies can both lead to HF, the mechanisms responsible for the development and progression of adverse ventricular remodeling are different. Whereas MI is characterized by ischemia-induced cardiomyocyte necrosis, leading to acute inflammation and replacement fibrosis (41), non-ischemic HCM, induced by chronic pressure overload due to hypertension or aortic valve stenosis in patients, does not result in significant cardiomyocyte death. Rather, non-ischemic HCM is characterized by pronounced cardiac hypertrophy, accompanied by blood vascular rarefaction and interstitial/perivascular fibrosis, also called ‘reactive fibrosis’ (41) related to chronic low-grade inflammation. Indeed, following a myocardial infarction, a so-called “replacement fibrosis” is occurring where extracellular matrix (ECM) replaces dying cardiomyocytes and muscle loss to maintain heart wall integrity, reinforcing the weakened myocardium. The resulting fibrotic scar is non-contractile, and its size, composition, and physical properties have major implications in the development of post-MI HF (42).

In an MI model induced by ligating the left coronary artery, post-MI survival was significantly improved in TNFR1KO but not TNFR2KO mice, while infarct size showed no change in either knockout strain. A loss of TNFR1 signal significantly ameliorated contractile dysfunction after MI, whereas a loss of TNFR2 signal significantly exaggerated ventricular dilatation and dysfunction (43). In WT mice, MI significantly increased both TNF and LTα levels in plasma, but in distinct temporal manners. While plasma TNF peaked 1 day after MI, and decreased toward baseline 3 days after MI, plasma LTα is significantly upregulated 3 days post-MI, and stayed elevated thereafter (44). Thus, TNF and LTα mediate post-MI cardiac dysfunction via TNFR1 stimulation, whereas TNFR2 activation is cardioprotective against ischemic injury. Thus, simultaneous inhibition of TNF and LTα or specific TNFR1 function blockade may represent superior cardioprotective approaches over general TNF activity suppression.

While TNF inhibitors have already proven successful in treatment of multiple inflammatory diseases presenting with end-stage fibrosis, there is evidence of potential additional uses of these inhibitors for treatment of tissue remodeling and fibrosis. Follow-up studies should focus on distinct pathogenic roles of TNFR1 versus TNFR2 and their selective inhibition as promising therapeutics for tissue-specific fibrosis.

## 3 LIGHT : HVEM/LTβR/DcR3

First described in 1998 by Mauri et al., LIGHT (homologous to Lymphotoxins, shows Inducible expression, competes with HSV Glycoprotein D for HVEM, a receptor expressed on T cells), also known as TNFSF14, HVEM-L (Herpesvirus Entry Mediator Ligand), or CD258, is a pleiotropic cytokine expressed primarily by activated T cells and dendritic cells with critical function in tissue fibrosis (2–4). Additionally, it is expressed in both soluble and membrane bound form (45). The receptors for LIGHT are LTβR (lymphotoxin-beta receptor), HVEM, and the Fas-ligand soluble receptor DcR3 (Decoy receptor 3), or TNFRSF3, TNFRSF14, and TNFRSF6B, respectively (45, 46). HVEM is a coreceptor for herpesvirus expressed broadly on structural and hematopoietic cells, including endothelial cells, adipocytes, macrophages, eosinophils, and T cells. HVEM-LIGHT interaction triggers intracellular signaling through NF-kB, stimulating cytokine production (47, 48). LTβR is necessary for lymphoid organ development and organization, cell proliferation, and apoptosis (49, 50). LTβR is similarly expressed on all the cell types mentioned except T lymphocytes. Most importantly, one or both receptors are expressed on the main cell types contributing to tissue remodeling and fibrosis: fibroblasts, smooth muscle cells, and epithelial cells. LIGHT is induced in several inflammatory diseases and autoimmune connective tissue disorders with fibroproliferative features, including asthma, idiopathic pulmonary fibrosis (IPF), atopic dermatitis (AD), SSc, eosinophilic esophagitis (EoE), and non-alcoholic fatty liver disease (NAFLD), and many others (51). Given its receptor expression and induction in diseased states, LIGHT is a key regulator of fibrosis in many organs.

In the lung, LIGHT has been shown to control fibrosis in models of severe asthma and models of IPF. In a house dust mite (HDM)-induced model of chronic asthma, Doherty et al. showed that pharmacological inhibition of LIGHT using an LTβR-Fc fusion protein significantly reduced smooth muscle hyperplasia, airway hyperresponsiveness, and lung fibrosis, which identified LIGHT as a target for asthmatic airway remodeling (52). A key finding from these studies in chronic asthma is that LIGHT acts on lung macrophages via LTβR, resulting in their accumulation and inducing their expression and subsequent release of TGFβ, the primary fibrotic cytokine. Furthermore, this work demonstrated that LIGHT can act on eosinophils via HVEM, significantly increasing their production of IL-13, another potent regulator of tissue remodeling that can synergize with TGFβ (52).

In an intratracheal bleomycin-induced model of PF, using both genetic deficiency of LIGHT and antagonistic blockade of LIGHT binding to HVEM and LTβR, Herro et al. demonstrated that LIGHT controls thymic stromal lymphopoietin (TSLP) expression (2). Recombinant LIGHT induces PF similarly to bleomycin induction, indicated by significant elevation in collagen deposition, αSMA accumulation around the bronchioles, and upregulation of mRNA transcripts for TGFβ and IL-13 (2). Additionally, abrogating LIGHT signaling significantly reduces TSLP and subsequent lung remodeling and fibrosis. Most notably, LIGHT not only synergizes with IL-13 and TGFβ, but it also acts *directly* on bronchial epithelial cells, inducing their expression of TSLP (2). This work suggests that LIGHT is playing a critical role in the fibrotic response in many pulmonary diseases.

A recent study by Qu et al. suggests that LIGHT plays a role in viral and bacterial sepsis induced acute respiratory distress syndrome (ARDS) (53). From a cohort of 280 patients, in the bacterial sepsis cases (n=189), significantly elevated LIGHT was associated with ARDS, higher Apache III scores, acute kidney injury (AKI), and acute hypoxic respiratory failure (AHRF). Given its profibrotic activity, LIGHT is likely a driver of worsened outcomes in bacterial sepsis.

LIGHT has been shown to be critical for fibrosis in SSc and AD. In a 2015 study, Herro et al. demonstrated that, similarly to its action in IPF, recombinant LIGHT given either subcutaneously or intratracheally induced features of SSc (3). LIGHT-deficient mice had significantly reduced skin fibrotic activity following bleomycin induction (3). Importantly, this work demonstrates that LIGHT acts through HVEM and LTβR expressed on epidermal keratinocytes to promote skin fibrosis. Similar to its activity in bronchial epithelial cells in IPF, LIGHT directly and indirectly (through synergy with TGFβ) controls TSLP expression in keratinocytes promoting skin fibrosis (3).

In another study by Herro et al., LIGHT signaling in keratinocytes was further investigated (4). Most notably, LIGHT-HVEM signaling in keratinocytes is required for HDM-induced AD, as it induced strong keratinocyte hyperplasia and periostin production (4). In LIGHT-deficient mice, clinical symptoms of AD were abrogated (i.e., skin eruption, bleeding, redness, and scaling). In addition, all type II cytokines were lower and TGFβ was nearly absent (4). Periostin, a characteristic feature of type II skin inflammatory disease, was also absent (4, 54). During HDM-induction, mice with a keratinocyte specific HVEM deletion phenocopied LIGHT-deficient mice. It was previously thought that periostin expression by keratinocytes was controlled by IL-13 and TGFβ, however, this work shows that it is directly controlled by LIGHT.

In a recent study, Ikawa et al. investigated the role of LIGHT, *in vitro*, on dermal fibroblasts from SSc patients (55). LIGHT was significantly elevated in bulk RNA-seq of diffuse cutaneous SSc skin biopsies (55). Conversely, HVEM was significantly decreased in mRNA from dermal fibroblasts. Given that HVEM deletion reduced dermal fibrosis to a greater extent than deletion of LTβR (3), this may suggest that HVEM profibrotic signaling in dermal fibroblasts is more potent that LTβR signaling. Further, *in vitro* recombinant LIGHT increases IL-6 expression and suppressed Th1 chemokine expression in diffuse cutaneous SSc dermal fibroblasts stimulated with IFNγ (55). This finding further asserts the role of LIGHT as a key regulator of fibrosis as the stimulation of IL-6 production by dermal fibroblasts would result in increased cellular activation, promoting both inflammation and fibrosis, and LIGHT suppression of Th1 chemokines is consistent with the fact that SSc is characterized by Th2/Th17 polarization.

In the liver, serum LIGHT was significantly increased in NAFLD patients, however, there were no differences found between NASH and simple steatosis (56). HVEM and LTβR mRNA expression was significantly increased in biopsied NAFLD tissue. LIGHT stimulated release of IL-8, a neutrophil chemokine, from human hepatoma cells (Huh7) was amplified by H_2_O_2_, which gives evidence to the role of oxidative stress in the pathogenesis of NAFLD. Genetic deletion of LIGHT resulted in reduced insulin resistance, hepatic steatosis, and expression of genes associated with NALFD to NASH transition in mice given a high-fat high-cholesterol diet for 16 weeks compared to controls (57). Furthermore, Liang et al. demonstrated that splenectomy improved liver fibrosis by decreasing the levels of serum LIGHT (58). Silencing of LTβR in macrophages *in vitro* resulted in decreased levels of fibrosis and αSMA in murine hepatic fibroblasts (JS1 cells) following treatment with recombinant murine LIGHT; and blocking TGFβ abrogated the effects of LIGHT *in vitro*. These findings suggest that LIGHT may induce TGFβ in hepatic macrophages, potentially driving liver fibrosis.

In the GI tract, LIGHT has been shown to play a role in esophageal fibroblast activation and differentiation into myofibroblasts, promoting inflammation and remodeling in EoE. In a study using cells from biopsied EoE tissue, LIGHT induced inflammatory gene transcription in fibroblasts and controlled tethering of eosinophils to fibroblasts via ICAM-1 (59). Additionally, subsequent treatment of TGFβ stimulated fibroblasts treated with LIGHT resulted in an inflammatory myofibroblast phenotype.

In the kidney, LIGHT has been implicated in controlling the development of renal fibrosis (60). LIGHT, HVEM, and LTβR were significantly elevated in sera and renal tissue from patients with chronic kidney disease (CKD). This was also reflected in unilateral ureteral obstruction (UUO)-induced mice. Notably, genetic deletion of LIGHT significantly reduced renal fibrosis in UUO mice, indicated by reduction in collagen deposition, αSMA and fibronectin expression, and reduced mRNA expression of TGFβ.

LIGHT has also been shown to play a significant role in connective tissue remodeling in primary acquired lacrimal duct obstruction (LDO), an idiopathic disease most common in the elderly. Bielecki et al. found that LIGHT and its receptors, HVEM and LTβR, are expressed on mononuclear cell infiltrates, endothelial cells, fibroblasts, and columnar epithelial cells of the lacrimal sacs (61). Notably, expression of LIGHT, HVEM, and LTβR were significantly correlated with fibrosis severity in the lacrimal sac walls.

**Figure 4:**
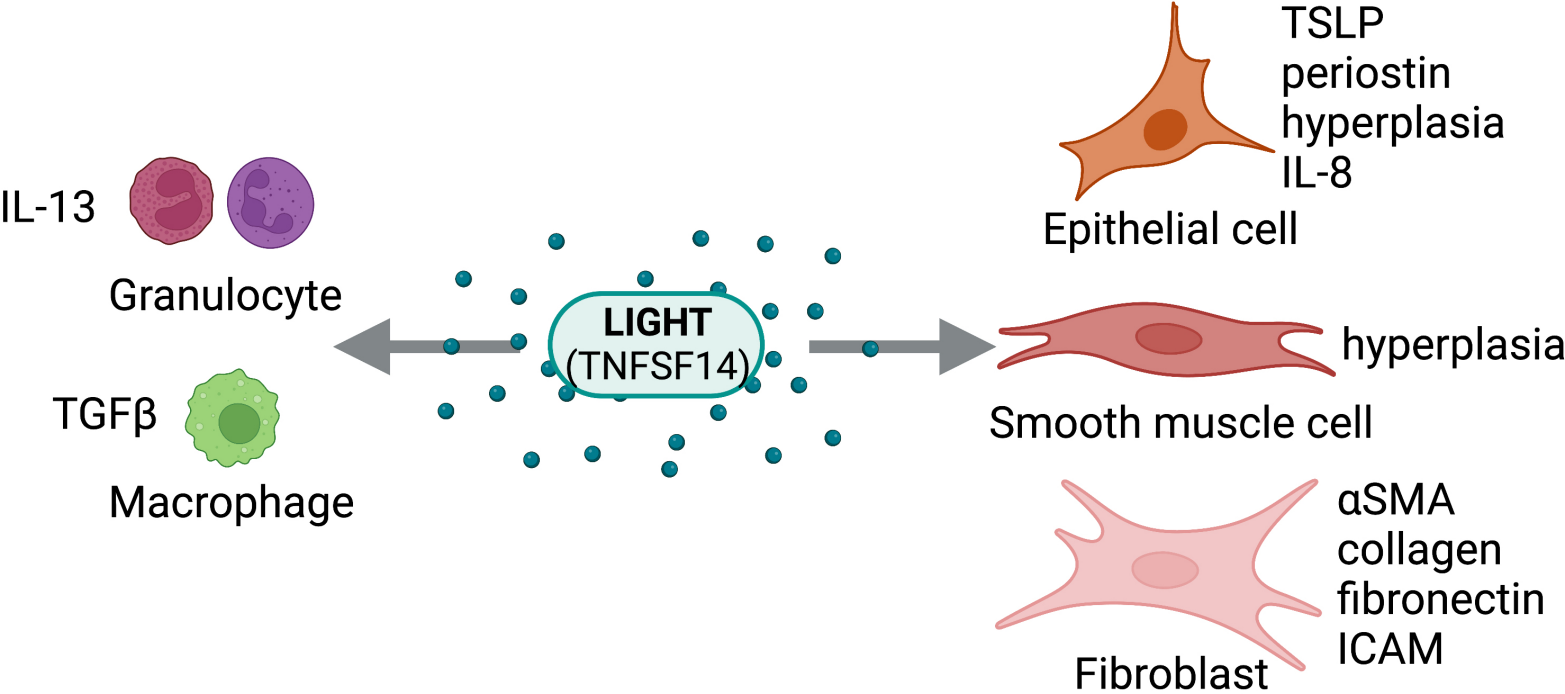
LIGHT Activity on Structural and Immune Cells. LIGHT (TNFSF14) can act directly on structural cells (epithelial cells, smooth muscle cells, fibroblasts) and immune cells (granulocytes, macrophages) to drive pro-fibrotic and anti-fibrotic effects. Figure was created with BioRender.com.

Given the findings from the studies described, LIGHT is a key regulator of fibrosis through both direct and indirect mechanisms (**Figure 4**). Additionally, signaling through either HVEM, LTβR, or both is necessary for LIGHT driven fibrosis. Thus, LIGHT is critical in the control of tissue remodeling and represents a promising therapeutic target for many fibrotic disorders.

## 4 TL1A : DR3/DcR3

The TNF-Like Ligand TL1A (TNFSF15) is a membrane-bound protein identified as a death receptor 3 (DR3) ligand in 2002 (62). It was found to be largely expressed on endothelial cells (62), though it has since been shown to be expressed cells of the immune system, including macrophages, plasma cells, and T lymphocytes (63). TL1A signals through DR3 (TNFRSF25) to induce the NF-kB pathway and acts as a costimulatory molecule for both activated T cells and ILC2s, leading to T cell expansion and the secretion of proinflammatory cytokines (62, 64). Additionally, TL1A signal can be neutralized by its soluble decoy receptor, DcR3 (TNFRSF6B) (65). The function of TL1A as a regulator of immunity and inflammation has led to numerous investigations of the role of TL1A expression in inflammatory disorders. Indeed, though its expression is usually systemically low, TL1A overexpression has been implicated in conditions such as inflammatory bowel disease and allergic inflammation of the lung and airways (63, 64, 66). Blocking the TL1A signaling pathway using an anti-TL1A antibody in colitis or asthma animal models has been shown to attenuate pathology and inflammation in these disease models (67), further highlighting TL1A as a promising marker for therapeutic strategies against inflammatory disease.

Additional studies indicate a potential for tissue-specific targeting of inflammation through the TL1A pathway. While it is initially expressed as a transmembrane protein, TL1A can be cleaved and expressed in a soluble form (62, 68). Transgenic mice expressing membrane-restricted TL1A were found to have elevated expression of several proinflammatory cytokines in the lung–indicating activation of both innate and adaptive immunity–whereas membrane-restricted TL1A in the small intestine primarily activated T cells, with a combination of soluble and membrane-bound TL1A required for severe bowel pathology (68). Of note, a consistently upregulated cytokine included IL-13, a major indicator of TL1A-driven inflammation through ILC2 activation (64, 66, 68, 69). In contrast, mice receiving soluble TL1A were found to have elevated expression of IL-13 and associated pathology of the bowel, but not in the lung (68). While potential therapies have been proposed that equally target both transmembrane and soluble forms (67), tissue-specific functional differences of TL1A indicate a potential for tissue-targeted therapies and may elucidate physiological implications of complete TL1A blockade.

Attenuating TL1A-mediated inflammation will have major implications for the prevention or treatment of chronic inflammatory complications leading to fibrosis. Disease models involving the gastrointestinal tract have frequently been used to study the role of TL1A in the development of fibrosis. While earlier studies indicated a role for TL1A in inducing intestinal inflammation (64, 66–69), recent studies have correlated elevated TL1A with intestinal fibrosis in mice, with affirming a direct role for TL1A-DR3 signaling on colonic fibroblasts and promoting intestinal fibrosis (70, 71). Treatment of Ulcerative colitis (UC) patients with anti-TL1A antibody has been shown to reduce expression of fibrotic pathway markers, including matrix metalloproteinases (*MMP7* and *MMP10*) (72), and treatment of the induced colitis mouse model with anti-TL1A antibody led not only to the reduction but also to the reversal of collagen deposition in mice with established fibrosis (73). Such results raise the potential for TL1A-DR3 signaling pathways to serve not only as a preventative therapeutic target, but also as a target for treatment of early fibrosis of the gastrointestinal tract.

As elevated TL1A expression has also been implicated in inflammation of the airway, recent studies have examined and affirmed the role of TL1A in promoting fibrosis of the lung. Treatment of mice with recombinant TL1A (rTL1A) has been shown to increase collagen deposition in the lung (5, 74), and treatment of human lung fibroblasts with rTL1A led to cell-specific proliferation and differentiation to myofibroblasts, as well as production of collagen (5). Blockade of TL1A-DR3 signaling in mice intranasally challenged with the HDM airway irritant resulted in decreased levels of collagen deposition compared to mice administered IgG control, which was accompanied also by a reduction in mucus production–an immediate threat to airway integrity driven by TL1A-driven IL-13 signaling (5, 74, 75). Interestingly, while a previous study suggested activation of both innate and adaptive immunity in the lung in the presence of overexpression of membrane-bound transgenic TL1A (68), rTL1A appears to promote mucus production in the absence of lymphocytes and independently of adaptive immunity (5). TL1A may therefore have temporal functions in the lung, signaling through innate and adaptive immune systems to differentially elicit an acute muco-secretory or chronic inflammatory response. Additional studies may enable targeted therapeutic strategies upon elucidating the different pathways leading to each pathology.

**Figure 5:**
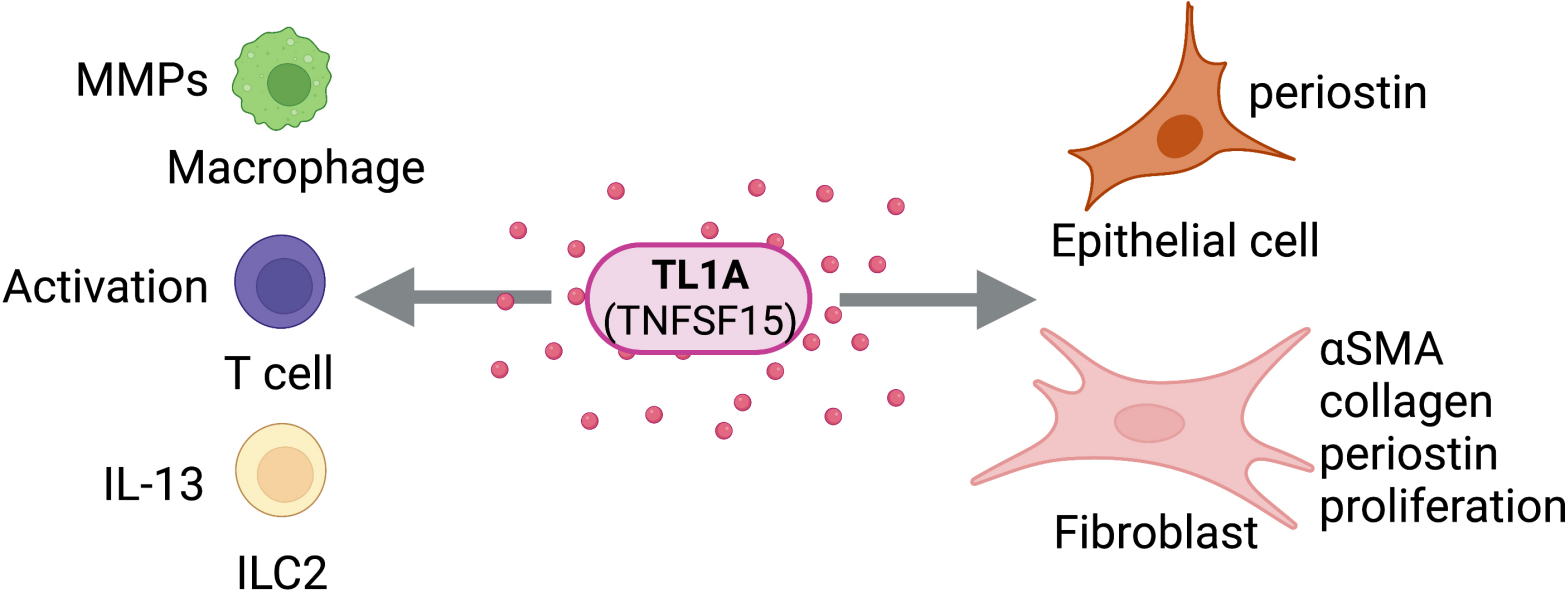
TL1A Activity on Structural and Immune Cells. TL1A (TNFSF15) can act directly on structural cells (epithelial cells, fibroblasts) and immune cells (macrophages, T cells, ILC2s) to drive pro-fibrotic and anti-fibrotic effects. Figure was created with BioRender.com.

As TL1A continues to be investigated as a potential therapeutic target for intestinal and lung inflammation, it is becoming increasingly clear that TL1A may be a systemically relevant target for fibrotic conditions in multiple body systems (**Figure 5**). TL1A was found to be upregulated in liver tissue in a recent study on mice with liver fibrosis, and TL1A transgenic mice exhibited higher levels of MMPs, collagen, and macrophage recruitment in the liver (76); however, studies linking TL1A with fibrotic disorders of the liver or other organ tissues are limited. TL1A has been established as a promising therapeutic target for inflammatory, fibrotic, and asthmatic disorders, pending further investigation of tissue-specific signaling pathways and mechanisms of action.

## 5 APRIL/BAFF : TACI/BCMA/BAFFR

**Figure 6:**
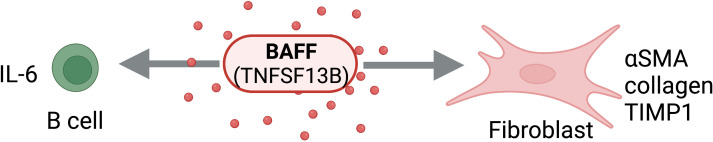
BAFF Activity on Structural and Immune Cells. BAFF (TNFSF13B) can act directly on structural cells (fibroblasts) and immune cells (B cells) to drive pro-fibrotic and anti-fibrotic effects. Figure was created with BioRender.com.

APRIL (TNFSF13), a proliferation-inducing ligand, and BAFF (also known as BLyS; TNFSF13B), a B lymphocyte stimulator, are two members of the TNF Superfamily (77–80). While BAFF can exist in membrane bound or soluble forms, APRIL exists only in soluble form (80–82). The ligands share two receptors of the TNFRSF family, TACI (TNFRSF13B) and BCMA (TNFRSF17), in addition to BAFF’s unique receptor, BAFF-R (TNFRSF13C) (83–88). Though APRIL and BAFF share receptors, their reported affinities differ significantly. On one hand, BAFF has high affinity for both BAFF-R and TACI, with a lesser affinity for BCMA; however, APRIL binds both TACI and BCMA with high affinity, but not at all to BAFF-R (89). All three receptors are expressed in circulating B lymphocytes, T lymphocytes, and monocytes (90). It also has been shown that TACI is expressed in macrophages, playing a role in M1 polarization and inflammation (91, 92). The roles of APRIL and BAFF in B cell maturation and differentiation have been thoroughly described (93–95); however, more recently, it has been shown that these TNFSF members contribute to pathogenic tissue remodeling and fibrosis (**Figure 6**).

Recent work has implicated elevated serum BAFF levels with elevated non-alcoholic steatohepatitis (NASH) severity. Miyake et al. showed that higher serum BAFF levels were associated with hepatocyte ballooning and advanced fibrosis in these patients, concluding that serum BAFF levels may be a useful tool for distinguishing NASH from simple steatosis (96). Similarly, in patients presenting with autoimmune hepatitis, BAFF levels were significantly higher in those with advanced fibrosis (a fibrosis score of F3 or higher) as opposed to those with less severe fibrosis (97). Concordantly, in an *in-vivo* study of nonalcoholic fatty liver disease (NAFLD), high fat diet-fed BAFF^-/-^ mice displayed reduced adipose tissue fibrosis and hepatic steatosis despite a lack of body weight reduction (98).

In patients with mixed connective tissue disease complicated by interstitial lung disease (ILD), serum levels of BAFF and APRIL were elevated compared to those presenting without ILD (99). However, only BAFF was elevated in the BALF, with no detected increase in APRIL, in a mouse model of bleomycin-driven PF. Additionally, BAFF inhibition in this PF mouse model by genetic ablation or neutralization by a soluble receptor, significantly reduced PF and IL-1β levels (100). Since BAFF is differentially upregulated in pulmonary fibrosis and correlative with disease severity, BAFF may represent an attractive therapeutic target, though further work examining the efficacy of pulmonary BAFF inhibition is needed.

Though BAFF is predominantly implicated in liver and lung fibrosis, research indicates that APRIL may play a role in atopic skin diseases. In studies of AD patients, elevated serum levels of APRIL, but not BAFF, are associated with disease severity (101, 102). High serum APRIL levels also strongly correlate with severity in pediatric atopic eczema (AE), both during flare and quiescence, implicating APRIL as a reliable marker of AE severity in children and highlighting APRIL as a promising target for treatment of AE (103). Further mechanistic and in-vivo studies are needed, however, to validate these correlations and understand the relationship between APRIL and fibrotic skin diseases.

Early work showed elevated serum BAFF levels in patients with SSc, with BAFF mRNA expression up regulated in affected skin of patients with early diffuse cutaneous SSc and B cell BAFFR expression increased in SSc patients as compared to healthy controls (104). Tight skin (TSK/+) mice are used to model development of skin fibrosis in SSc. In these TSK/+ mice, serum BAFF levels are significantly elevated and BAFF antagonist inhibits the development of skin fibrosis and suppresses fibrogenic cytokine production, such as IL-6 and IL-10 (105). Work by Matsushita et al. showed that increased levels of APRIL and BAFF are mutually exclusive in SSc patients, with high APRIL levels indicating pulmonary involvement and high BAFF levels serving as a marker for severe skin fibrosis (106). Opposing these findings, Bielecki et al. found significant association between increased production of APRIL by PBMC and greater skin fibrosis, in addition to pulmonary involvement and increased anti-topo I antibodies (107). Further work must be done to elucidate the distinct and overlapping roles of BAFF and APRIL in SSc pathogenesis.

Recent studies also explore the pathogenic role of B cells and BAFF in fibrosis and systemic sclerosis. B cells stimulated with BAFF can upregulate profibrotic markers such as collagen, *αSMA*, and *TIMP1* in SSc human dermal fibroblasts (108). BAFF inhibition can attenuate fibrosis in a bleomycin-induced mouse model SSc with reduction of fibrotic IL-6 producing Beffs, but not regulatory IL-10 producing Bregs (109). The exact contribution of B cells to fibrotic processes, however, remains unclear and needs further investigation.

BAFF and APRIL, along with their shared and distinct receptors, remain promising therapeutic targets for inflammatory and fibrotic disorders; however, further studies must be done to understand disease and tissue-specific signaling of these molecules.

## 6 RANKL : RANK/OPG

The receptor activator of nuclear factor kappa-B ligand (RANKL) is a TNFSF member (TNFSF11) most well-known for its role in osteoclastogenesis, maintaining bone homeostasis, and degradation of ECM in bone tissue (110). The effects of RANKL are mediated by its binding to its specific, high affinity receptor, the receptor activator of nuclear factor kappa-B (RANK; TNFRSF11A). RANK it is expressed on cells of macrophage/monocyte lineage, which includes bone osteoclasts (111). When RANKL binds RANK, it activates and differentiates osteoclasts and degrades the extracellular bone matrix (110). RANKL also has a decoy receptor, osteoprotegrin (OPG; TNFRSF11B), which is soluble protein expressed mostly by osteoblasts and an inhibitor of osteoclast activation, counteracting bone resorption (110, 111). OPG binds RANKL, limiting RANKL/RANK binding and thus preventing activation and differentiation of osteoclasts. Under normal physiological conditions, the RANKL/RANK/OPG axis is vital for regulating bone turnover and maintaining bone homeostasis. However, in a disease state, the axis can become unbalanced and drive fibrosis. Elevated levels of OPG can inhibit ECM degradation by blocking RANKL-RANK interactions, leading to the buildup of ECM characteristic of fibrotic diseases.

RANKL is upregulated in multiple fibrotic liver diseases, indicating it may play a key role in pathogenesis. Primary biliary cholangitis (PBC) is a disease characterized by injury to the small intrahepatic bile ducts. PBC patient cholangiocytes express elevated levels and RANKL and RANK, which can be associated with disease severity (112). Although the exact role of the RANKL/RANK axis in PBC is unclear, activating this signaling pathway inhibits the proliferation of cholangiocytes. The RANKL/RANK axis may have a protective role in PBC and represents a strong potential target for treating PBC (112). RANKL and OPG expression are both significantly elevated in serum of patients with liver cirrhosis, a disease in which low bone density and bone mass loss can be potential complications. Fabrega et al. suggest that RANKL is stimulated by disease-associated pro-inflammatory cytokines, causing enhanced osteoclast formation, and that the elevated OPG may be an attempt to counteract the bone loss caused by this osteoclast formation (111).

The RANKL-RANK-OPG axis has been implicated in fibrotic lung disease, though further work is needed to better understand its exact role. In silicosis, a type of pulmonary fibrosis caused by silica inhalation, silica activates lung macrophages and bone osteoclasts via RANKL and TLR4 signaling pathways. This process is thought to increase proteolytic activity of macrophages with a TRAP+ and high MMP-12 phenotype, which may lead to elastin degradation and promote lung fibrosis (113). In a mouse model of silicosis, work by Jin et al. showed that treatment with the naturally occurring tetrapeptide N-acetyl-seryl-aspartyl-lysyl-proline (Ac-SDKP) reduces RANKL signaling in macrophages and RANKL-induced osteoclast differentiation, highlighting a potential therapeutic role for blocking RANKL-signaling via Ac-SDKP (113). In cystic fibrosis (CF), CF-related bone disease, characterized by low bone mineral density and increased risk of fracture, is a rising cause of morbidity (114). Human osteoblasts with a CFTR mutation showed RANKL overexpression and decreased OPG production. This combination shows an increased RANKL-to-OPG ratio, which can drive decreased bone resorption that leads to CF-associated bone loss (114).

The involvement of OPG pathways in the pathology of cardiac fibrosis is evidenced by several studies. In addition to MI HF, two other forms of cardiac fibrosis may occur and often co-exist: interstitial and perivascular fibrosis. While not necessarily resulting of cardiomyocytes death, these may result in pressure overload or metabolic dysfunction, due to endothelial and immune change in response to hypoxia. Both reactive forms of fibrosis lead to accumulation of fibrous tissue in the perivascular space of coronary arteries and drive cardiac muscle bundle thickening.

Ageing OPG-KO mice developed less interstitial cardiac fibrosis and concomitant activation of MMP-2 (115), which is known to be associated with fibrosis resolution (116), tissue inhibitors of MMP-1, -2 and the inactivation of procollagen α1 synthesis, while developing cardiac left ventricle hypertrophy and significant loss of contractile function. Moreover, in OPG-KO mice, cardiac hypertrophy following Ang-II-induced hypertension is significantly increased, while less interstitial fibrosis and pro-collagen-α1 mRNA expression but increased numbers of apoptotic cells is observed (117). Moreover, blocking IgE-FcERI pathways in TAC- or Ang-II-induced-HCM alleviate pathological heart remodeling and dysfunction. Indeed, *in vitro* IgE treatment of neonatal cardiomyocytes or cardiac fibroblast induced hypertrophy and activation with matrix protein production and OPG gene expression, respectively (118). In human, OPG levels are significantly higher in patients with chronic fibrosis after myocardial infarction than those with no fibrosis, associated with aortic stenosis severity and poorer prognosis (119). In addition, higher OPG levels were associated with increased left ventricular mass index and myocardial stiffness in patients suffering of HF with preserved ejection fraction (120). Collectively, these data suggest a critical role for OPG/RANK/RANKL axis in pathological cardiac remodeling.

The RANKL-RANK-OPG axis represents a potential avenue to develop novel diagnostics and therapeutics for fibrotic disease. OPG expression is upregulated in multiple fibrotic tissues, highlighting the RANKL-OPG interaction as a promising candidate to include in a biomarker panel to diagnose fibrosis (110, 121). Wang et al. showed RANKL mutants that can bind RANK but not OPG, which could be a strong therapeutic tool that warrants further investigation. This therapeutic works by allowing the RANKL-RANK interaction to occur, while limiting obstruction from elevated OPG levels. Future studies should be performed to further explore the therapeutic potential of targeting the RANKL-RANK-OPG signaling axis to treat fibrotic diseases.

## 7 GITRL : GITR

Glucocorticoid-induced TNF receptor family related protein (GITR), also known as TNFRSF18, is a type I transmembrane protein which is constitutively expressed by Tregs at a high level and by resting CD25^-^CD4^+^ T cells at a lower level, though expression is markedly increased following T cell activation (122). In addition to activated effector T lymphocytes, GITR may also be found on other activated immune cells such as NK cells and neutrophils (123–125). GITR is activated by its ligand GITRL (TNFSF18), which is mainly expressed on B cells, DCs, macrophages and endothelial cells (126, 127). GITR possesses an intracellular domain bearing significant homology to those of fellow TNFRSF members CD27, OX40, and 4-1BB, all of which act as powerful costimulatory signals for T cells (128–130). GITR itself is no exception in this regard as it functions as a powerful costimulatory molecule for all T cell subpopulations (124). Stimulation of CD25^-^CD4^+^ T cells with agonistic anti-GITR antibody (DTA-1) results in promotion of proliferative responses, cytokine production, and expression of activation antigens (131). Interestingly, while GITR stimulation of CD25^+^CD4^+^ T cells (Tregs) results in increased proliferation, it also leads to abrogation of Treg mediated suppression (122, 132).

This relationship between GITR and Tregs was further investigated by Kim and Youn through *in-vitro* cocultures of arthritic mouse derived fibroblast-like synoviocytes (FLS) and Tregs to simulate inflamed synovia. In Tregs, GITRL-expressing FLS caused a reduction in the expression of Foxp3, the master transcription factor which drives Treg development and functionalization (133). Constitutive expression of GITR on Tregs has been implicated in generating signals which dampen Foxp3-mediated suppressive action and decrease expression stability (134). Further, anti-GITR mAb (DTA-1) treated Tregs resisted FLS-induced Foxp3 downregulation, which supports the evidence that this Foxp3 downregulation occurs due to GITR signaling (132, 133). This functional downregulation of Tregs subsequently creates a pro-inflammatory environment which can develop into fibrosis if unresolved; however, further *in vivo* studies investigating this phenomenon in fibrotic models are needed to unravel the relationship among GITR, Tregs, and fibrosis.

In a study by Chen et al., they used a co-culture model of bleomycin treated MLE-12 cells (immortalized mouse lung type II epithelial cell line) and human menstrual blood-derived mesenchymal stem cells (MenSC). MenSC were able to significantly reduce bleomycin induced cell injury and inhibit bleomycin induced epithelial to mesenchymal transition (EMT) (135). Bleomycin treated MLE-12 cells cocultured with MenSC showed significant downregulation of GITR compared to bleomycin treated MLE-12 cells cultured alone (135). Immunophenotyping of 79 patients of varying degrees of liver cirrhosis found significant upregulation GITR, CD40L, and OX-40 in patients with cirrhosis as compared to healthy controls (136). Combined, these findings strongly suggest that GITR signaling plays a role in promoting fibrosis, and thus could serve as a potential therapeutic target.

The direct role of GITR signaling in the context of fibrosis is perhaps best illustrated by the findings of Cuzzocrea et al., where bleomycin treated GITR^-/-^ mice displayed significantly diminished histological signs of lung injury as compared to GITR^+/+^ mice in a bleomycin model of pulmonary fibrosis (137). Further, cotreatment of GITR^+/+^ mice with bleomycin and a GITRL binding Fc-GITR fusion protein resulted in a phenotype resembling that of GITR^-/-^ mice, suggesting that these results are indeed due to disruption of GITRL/GITR signaling. Analysis of BALF also shows that GITR^-/-^ BALF has a lower degree of cellularity compared to that of GITR^+/+^ mice. Histological findings correlate areas of lung injury with granulocyte infiltration and show significant reduction of myeloperoxidase (MPO) activity in both GITR^-/-^ and Fc-GITR treated GITR^+/+^ mice as compared to GITR^+/+^ mice (137). Another potential contributing factor for the GITR^-/-^ phenotype is the responsiveness of fully polarized Th2 cells and lack of responsiveness of fully polarized Th1 cells to GITR stimulation, as shown by *in vitro* studies conducted by Motta et al. (138). This distinction is especially relevant as Th2 cytokine signaling is known to play a central role in the perpetuation of fibrotic processes (139). The reduction in leukocyte recruitment coupled with an absence of the GITR stimulated pro-Th2 signaling may generate a relatively anti-inflammatory and anti-fibrotic environment, thus possibly explaining the above findings. Though recent studies strongly imply GITR’s involvement in fibrosis, additional studies are needed to identify the exact mechanisms responsible.

## 8 OX40 : OX40L

OX40 (also known as TNFRSF4 or CD134) was originally found to be a marker of T-cell activation, as it is only expressed transiently by effector T-cells following antigen binding of the T-cell receptor (140). Expression of OX40 ligand (OX40L, also known as TNFSF4 or CD252), is largely limited to professional antigen presenting cells such as dendritic cells or activated B cells (141, 142). OX40-OX40L signaling is known to modulate activated T-cell activity through co-stimulatory signaling (143). More specifically, OX40 signaling promotes activated T-cell survival, proliferation, and cytokine secretion (144). OX40-OX40L interactions are thus unsurprisingly implicated in various autoimmune diseases such as SSc or systemic lupus erythematosus (SLE) (145, 146). However, recent studies suggest that OX40-OX40L may play direct role in causing or promoting fibrosis.

The impact of OX40L expression within the context of a potential new animal model of pulmonary arterial hypertension (PAH) was investigated in a study by Rabieyousefi et al. PAH refers to a category of disease which typically presents clinically with severe pulmonary hypertension and presents histopathologically with pulmonary arteriole occlusion, medial muscular hypertrophy, and intimal fibrosis (147). Spontaneous PAH onset was stably identified in transgenic C57BL/6 (B6) mice which overexpressed OX40L (B6.TgL). Curiously, this spontaneous onset of PAH was found to be dependent on the presence of OX40L specifically in the B6 genetic background. Pathological manifestations in the colon and lung were observed in transgenic mice with constitutive OX40-OX40L signaling with a B6 genetic background but not in mice with a BALB/c genetic background. These findings aptly point out that the importance of OX40 signaling in fibrosis may become even further enhanced when in the presence of certain predisposing genetic factors.

In a study by Elhai et al., *in vivo* blockade of OX40L in murine models of SSc prevented the inflammation-driven fibrosis, fibrosing alveolitis, and lung vessel remodeling normally associated with this model (148). Additionally, soluble OX40L levels were significantly higher in SSc patient serum samples compared to healthy controls, especially in patients with diffuse cutaneous SSc, the most severe presentation of SSc. Elevated OX40L serum levels at baseline were highly predictive for worsening of dermal and lung fibrosis, indicating that OX40L may serve as a potential biomarker for fibrosis. Current work further suggests OX40L expression may impact development and progression of fibrosis in a variety of different tissues and disease states.

In the skin, the same study by Elhai et al. also found that OX40L lacking mice were protected against bleomycin induced dermal fibrosis, as evidenced by decreased dermal thickness, hydroxyproline content, and myofibroblast count compared to OX40L expressing mice (148). Blockade of OX40L with anti-OX40L monoclonal antibodies even caused regression of established dermal fibrosis in the bleomycin mouse model. Histological examination of biopsied fibrotic skin from SSc patients showed the presence of OX40L staining in cells positive for both CD90 and αSMA, suggesting OX40L is also expressed by fibroblasts and myofibroblasts in fibrotic skin.

OX40 signaling may also play a central role in the process of atherosclerosis. Atherosclerosis-prone receptor deficient mice (LDLR^-/-^) were placed on a Western-type diet for 10 weeks to induce atherosclerotic lesions in the aorta and aortic arch before being placed on a chow diet and receiving concurrent treatment with anti-OX40L antibody or PBS (149). While the decrease in dietary lipids alone did improve lesion stability, lesion regression did not occur unless anti-OX40 antibody was also administered. Lesion regression is likely largely due to the loss of Th2 promotion normally caused by OX40-OX40L signaling. This reduction of Th2 polarization is supported by flow cytometry showing a significant reduction in GATA-3 positive cells within the CD4 positive T-cell population. OX40-OX40L signaling disruption represents a potential therapy for established atherosclerotic lesions.

Interference of OX40-OX40L signaling presents as an attractive avenue of potential prophylaxis and/or therapy for a variety of fibrotic diseases including systemic sclerosis, pulmonary arterial hypertension, dermal fibrosis, and atherosclerosis. Soluble OX40L levels also possesses potential as a biomarker for fibrosis and is predictive of worsening dermal and lung fibrosis in systemic sclerosis patients. Recent studies have also demonstrated that OX40L expression extends beyond just professional antigen presenting cells to also include structural cells such as fibroblasts and myofibroblasts. Future studies should seek to further examine the impact of OX40-OX40L signaling in fibrotic diseases beyond the canonical co-stimulation of T-cells through antigen presenting cells.

## 9 CD70 : CD27

CD70 (TNFSF7) is the single natural cognate ligand for the transmembrane glycoprotein CD27 (TNFRSF7). CD70 is expressed on antigen presenting cells and select other cell types like fibroblasts (150), while expression of CD27 is found on specific subsets of B and T lymphocytes, NK cells and hematopoietic stem cells (151). The CD27:CD70 costimulatory pathway has previously been well characterized as an important axis for signal transduction among T and B cells (152, 153). This pathway is important for priming naïve T cells and promoting their survival, which directly affects the formation of effector and memory T lymphocytes. The activation of T cells leads to MMP-mediated proteolytic cleavage of CD27 from the cell surface, resulting in secretion of functionally active soluble CD27 (sCD27) (154–156). Thus, CD27 is expressed on naïve T cells and memory CD4+ lymphocytes, but not on activated effector cells (152, 157). Concordantly, expression of CD27 is absent from naïve B cells, but upregulation occurs in activated and memory B lymphocytes (158). The CD70:CD27 cognate interactions in the B cell compartment are necessary for promotion of T cell dependent activation of B cells, the formation of germinal centers, as well as subsequent expansion and differentiation of B cells into plasma cells (158–161). While CD70:CD27 interactions have been thoroughly recognized as providing stimulatory signals for T and B cell activation, the involvement of this axis in fibrosis remains unclear.

Recent research investigating the interaction of CD70 on fibroblasts proposes CD70 as a possible target in fibrotic diseases. Tran-Nguyen et al. demonstrated that CD70 agonists, including T-cell–derived sCD27, were potent inhibitors of fibroblast extracellular matrix protein production such as collagen and fibronectin (162). This work highlights CD70:CD27 axis modulated T cell-fibroblast interactions, linking T cell response to fibrogenesis. In an acute inflammatory state, high CD27 expression and sCD27 secretion by activated CD4 T cells drive elevated fibroblast CD70 activation, consequently inhibiting fibroblast ECM production. Conversely, chronic inflammation is predominated by highly differentiated CD4 T-cell effector-memory (Tem) that do not express or secrete CD27, resulting in enhanced fibrosis. Though these studies used in-vitro and ex-vivo modeling to suggest that stimulation of fibroblast CD70 with an agonist could be a strategy to target fibrosis in chronic disease, further development of in-vivo modeling is needed to confirm the potential for targeting CD70 as an antifibrotic treatment.

Mature CD20+ CD27+ B cells were found to be increased in chronic renal nephritis biopsies in the form of intrarenal lymphoid follicle-like structures and their levels are correlative with disease severity (163). Though double labeling for CD27 and CD20 demonstrated a significant mature memory B cell population, whether these CD20+ CD27+ B cell-dominated structures represent a harmful or potentially beneficial occurrence for progression of renal diseases remains unclear. Future loss of function studies must be done to understand the potential contribution of these mature B cells to fibrosis development.

Doi et al. observed a depletion of CD27+ B cells in cirrhotic patients compared to healthy controls (164). In addition, the loss of this population was accompanied by impairment of activation, T-cell allostimulation, TNF-beta secretion and IgG production. Supporting these findings, Chang et al. also showed a lack of CD27+ memory B cells in cirrhotic patients (165). Further, in this limited pool of CD27+ B cells from cirrhotic patients, there was a statistically significant increase of the pro-apoptotic CD95 (Fas/TNFRSF6). They concluded that elevated sensitivity to Fas-mediated apoptosis, directed by increased exposure surface FasL and endotoxin, was likely driving the depletion of CD27+ memory B cells and contributing to systemic infection risk. To resolve the role of these B cells in cirrhosis, further mechanistic and in-vivo studies are needed to clearly define any causal relationships between these phenomena.

In an in-vivo experimental autoimmune myocarditis (EAM) model, activated cardiac NK cells expressed CD27 and depletion of CD27+ NK cells during EAM resulted in increased disease severity, including elevated fibrosis and an influx of cardiac infiltrating eosinophils (166). *In vitro*, CD27+ NK cells limited infiltration of eosinophils both directly, through induction of eosinophil apoptosis, and indirectly, via alteration of eosinophil-related chemokines by cardiac fibroblasts. Thus, while this work proposes a pathway of NK cell-driven eosinophilic regulation, further confirmation in human models is needed to determine therapeutic potential of targeting CD27+ NK cells.

Serum levels of soluble CD27 positively correlated with disease severity in SSc biopsies, highlighting the potential for targeting the CD27:CD70 axis (161). Thus, skin tissue expression of CD27 and serum levels of sCD27 may be useful diagnostic markers for SSc. Similarly, Luo et al. emphasized epigenetic involvement in disease pathogenesis and highlighted the differential expression of CD70 in SSc patients, which is associated with disease severity (167). These studies remain correlative, however, and require in-vivo validation to determine the contribution of the CD27:CD70 axis in SSc development.

## 10 TRAIL : DR4/DR5/DcR1/DcR2

Tumor necrosis factor-related apoptosis-inducing ligand (TRAIL), also known as TNFSF10, Apo-2 ligand, or CD253, is a Type II membrane protein bearing high levels of homology to Fas ligand (168). TRAIL is primarily expressed by lymphocytes, monocytes, and natural killer cells (169–171). Though membrane bound, TRAIL can also undergo proteolytic cleavage and be released in a soluble form which then goes on to bind one of five different receptors. The canonical TRAIL signaling pathway involves its binding to either of two death domain containing death receptors, death receptor 4 (DR4, also known as TRAIL-R1 or TNFRSF10A) (172) or death receptor 5 (DR5, also known as TRAIL-R2 or TNFRSF10B) (173), thus prompting receptor homotrimerization along with the recruitment of the adaptor protein Fas-associated death domain (FADD), resulting in the formation of death-inducing signaling complex (174). FADD then recruits and activates procaspase-8 and downstream signaling events ultimately lead to apoptosis (174). TRAIL may also bind to either of two membrane decoy receptors, decoy receptor 1 (DcR1, also known as TRAIL-R3 or TNFRSF10C) which completely lacks a death domain (175) or decoy receptor 2 (DcR2, also known as TRAIL-R4 or TNFRSF10D) which possesses a truncated non-functional death domain (176). Finally, the secreted glycoprotein and TNF superfamily member OPG serves as a soluble decoy receptor for TRAIL, albeit with slightly reduced affinity compared to DcR1 and DR5 (177). Mice possess two TRAIL decoy receptors and a single TRAIL death receptor (mDR5) which bears greater similarity to human DR5 than to human DR4 (178).

Though TRAIL is mainly known for its ability to selectively induce apoptosis in cancer and transformed cells while sparing normal cells (179, 180), its potential beyond the treatment of cancer has gained growing attention in recent years. TRAIL has robust applications in the context of liver fibrosis. Activated hepatic stellate cells (aHSCs) are the cell type primarily responsible for liver fibrosis and were found to have increased expression of DR4 and DR5 (181). This upregulation of DR4 and DR5 suggests that TRAIL may be capable of selectively targeting aHSCs to effectively treat liver fibrosis. Indeed, systemic administration of PEGylated TRAIL was found to ameliorate carbon tetrachloride (CCl_4_) induced liver fibrosis and cirrhosis in rats, as measured by reductions in αSMA and collagen deposition, via selective killing of aHSCs without any off-target apoptosis. The remarkable specificity of this preparation is noted by the authors to contrast with the recombinant TRAIL formulations and TRAIL agonists used in cancer research. This suggests that the design of anti-fibrotic TRAIL/TRAIL agonist formulations will require additional considerations to limit off-target effects and perhaps include a mechanism to eventually suspend apoptosis to allow for tissue healing. This work also emphasizes the importance of screening potentially targetable cell types in a fibrotic disease for TRAIL sensitivity, as aHSCs are known to not undergo spontaneous apoptosis and resist a myriad of proapoptotic stimuli (182).

The efficacious results of this approach are also demonstrated by Park et al. in the investigation of engineered TRAIL treatment in SSc. Dermal myofibroblasts are the principal cell type responsible for fibrosis in scleroderma and thus present as an obvious potential therapeutic target. RNA-seq data from skin biopsies of SSc patients revealed greatly upregulated DR4 and DR5 mRNA expression in addition to other typical fibrogenic components (183). *In vitro* studies of primary human dermal fibroblasts show that TGF-β1, the cytokine which induces dermal myofibroblasts transdifferentiation, also significantly induces both mRNA and protein expression of DR4 and DR5. Treatment with engineered TRAIL not only selectively killed dermal myofibroblasts but was even able to reverse established skin fibrosis to near-normal architecture in both an inducible (bleomycin) and genetic (Tsk-1) mouse model of SSc.

It is also becoming increasingly evident that TRAIL can play a major role in fibrotic diseases by affecting inflammatory processes. In investigating the role of TRAIL in the *A fumigatus* mouse model of EoE, TRAIL was significantly upregulated in EoE patients as compared to their healthy counterparts (184). TRAIL is also known to upregulate expression of the E3 ubiquitin-ligase midline-1 (MID-1), whose downstream signaling events promote inflammation through inhibition of protein phosphatase 2A (PP2A) activity. TRAIL deficient mice treated with MID-1 targeted small interfering RNA (siRNA) displayed reduced esophageal eosinophil and mast cell counts along with protection against esophageal circumference enlargement and collagen deposition. Additionally, TRAIL was found to be necessary for the upregulation of four key cytokines implicated in EoE pathogenesis: CCL11, CCL24, TGFβ, and TSLP. It was also discovered that the production of IL-5, which is the major driver of allergen induced-EoE, and IL-13, which plays a supporting role to IL-5, are TRAIL dependent. Histological findings also demonstrated that TRAIL expression is required for esophageal remodeling in this model of EoE.

TRAIL exemplifies the diversity of functions found within the TNF superfamily and shows great promise as a potential therapy for several fibrotic diseases because of it. Engineered TRAIL has demonstrated excellent selectivity and efficacy in killing target cell types in multiple fibrotic diseases, leading to amelioration and sometimes even reversal of fibrosis in animal models. Notably, TRAIL can induce apoptosis in cell types which are known to be particularly resistant, such as aHSCs and MFBs. TRAIL is therefore an excellent therapeutic candidate in fibrotic diseases where the primary driver of fibrosis is TRAIL sensitive or could reasonably be induced to become TRAIL sensitive. However, there is a need for further investigation into screening potential target cell types for TRAIL sensitivity and signaling pathways which affect TRAIL, DR4, and DR5 expression. Finally, the recent findings showing TRAIL’s involved role in the various inflammatory processes leading to EoE highlights the need explore TRAIL signaling beyond the canonical apoptotic paradigm with emphasis on non-canonical TRAIL signaling.

## 11 Concluding remarks

Although TNFSF members were first identified as mediators of inflammation, apoptosis, and cell survival, our lab and others have recently implicated these members as drivers and regulators of fibrosis and tissue remodeling in many diseases and across numerous organs. Based on the growing literature, select TNFSF members are up regulated in fibrotic disease states in humans. These TNFSF members represent potential therapeutic targets for improving fibrotic disease outcomes and novel biomarkers for tracking disease progression in minimally invasive manners. TNF inhibitors are already approved and efficacious for treatment of diseases presenting in end stage fibrosis, including UC, rheumatoid arthritis, psoriatic arthritis, ankylosing spondylitis, juvenile idiopathic arthritis, Crohn’s disease, plaque psoriasis, juvenile idiopathic arthritis, and non-radiographic axial spondyloarthritis. Currently ongoing clinical trials at many stages of development for other TNFSF inhibitors, described in **Table 2**, indicate a potential for targeting additional members in fibrotic disorders. Given current research on TNFSF members and fibrosis, in patients with scarring and tissue remodeling, blocking select TNFSF members by either neutralizing reagents or by antibodies to their receptors may represent a therapeutic benefit compared to presently available treatments which only slow disease progression as opposed to reversing fibrosis.

**Table 2:** Clinical trials targeting TNF superfamily members to treat fibrotic disease.

NCT Number	Target	Therapeutic	Title	Conditions	Interventions	Sponsor/Collaborators	Phases	Status	Study Results	Results First Posted
NCT03747068	TNF	anti-TNF mAb	The Influence of Biological Treatment on the Short-Term Complications of Surgery in Patients With Inflammatory Bowel Disease.	Ulcerative Colitis Anti TNF Therapy Ileal Pouch Anal Anastomosis (IPAA)	Drug: Anti-TNF Drug	HaEmek Medical Center, Israel Mount Sinai Hospital, Canada University of Toronto		Completed	No Results Available	
NCT03180957	TNF	anti-TNF mAb	Repurposing Anti-TNF for Treating Dupuytren’s Disease	Dupuytren’s Disease	Drug: Adalimumab Drug: Saline	University of Oxford Department of Health, United Kingdom Wellcome Trust 180 Therapeutics LP	Phase 2	Completed	No Results Available	
NCT00385086	TNF	anti-TNF mAb	Lumbar Spinal Fibrosis and TNF Alpha Inhibition	Post Operative Sciatica by Lumbar Spinal Fibrosis	Drug: TNF blocker Drug: Placebo	Assistance Publique - Hôpitaux de Paris	Phase 3	Completed	No Results Available	
NCT00063869	TNF	soluble TNFR2-Fc fusion protein	Study Evaluating the Safety and Efficacy of Etanercept in Patients With Idiopathic Pulmonary Fibrosis	Pulmonary Fibrosis	Drug: Etanercept	Wyeth is now a wholly owned subsidiary of Pfizer	Phase 2	Completed	No Results Available	
NCT05323110	LIGHT	anti-LIGHT mAb	Study of Intravenously Administered Anti-LIGHT Monoclonal Antibody CBS001 in Healthy Volunteers	Chronic Inflammatory Disease	Drug: CBS001 Drug: Placebo	Capella Bioscience Ltd Centessa Pharmaceuticals plc	Phase 1	Recruiting	No Results Available	
NCT05288504	LIGHT	anti-LIGHT mAb	A Study to Evaluate the Safety and Efficacy of AVTX-002 for the Treatment of Poorly Controlled Non-Eosinophilic Asthma.	Non-Eosinophilic Asthma	Drug: AVTX-002 Drug: Placebo	Avalo Therapeutics, Inc.	Phase 2	Recruiting	No Results Available	
NCT04412057	LIGHT	anti-LIGHT mAb	Clinical Trial to Evaluate CERC-002 in Adults With COVID-19 Pneumonia and Acute Lung Injury	COVID-19 Pneumonia Acute Lung Injury ARDS	Drug: CERC-002 Drug: Placebo	Aevi Genomic Medicine, LLC, a Cerecor company Avalo Therapeutics, Inc.	Phase 2	Completed	Has Results	4-Mar-22
NCT03169894	LIGHT	anti-LIGHT mAb	Evaluation of the Safety, Tolerability, and Efficacy of MDGN-002 in Adults With Moderate to Severe Active Crohn’s Disease or Ulcerative Colitis	Crohn Disease Ulcerative Colitis	Drug: MDGN-002	Aevi Genomic Medicine, LLC, a Cerecor company Avalo Therapeutics, Inc.	Phase 1	Terminated	No Results Available	
NCT01552681	LTβR	anti-LTβR Fc fusion protein	Baminercept, a Lymphotoxin-Beta Receptor Fusion Protein, for Treatment of Sjögren’s Syndrome	Primary Sjögren’s Syndrome	Biological: Baminercept Other: Placebo	National Institute of Allergy and Infectious Diseases (NIAID) Autoimmunity Centers of Excellence Biogen	Phase 2	Terminated	Has Results	23-Mar-16
NCT05354349	TL1A	anti-TL1A mAb	Bioavailability of SC Formulation and Japanese Ethnobridging Study for PRA023	Healthy	Drug: PRA023 IV Low Dose Drug: PRA023 SC Drug: Placebo IV Drug: Placebo SC Drug: PRA023 IV High Dose	Prometheus Biosciences, Inc. altasciences	Phase 1	Active, not recruiting	No Results Available	
NCT05270668	TL1A	anti-TL1A mAb	Phase 2 Safety and Efficacy Study of PRA023 in Subjects With Systemic Sclerosis Associated With Interstitial Lung Disease (SSc-ILD)	Diffuse Cutaneous Systemic Sclerosis Interstitial Lung Disease	Drug: PRA023 IV Device: Companion diagnostic (CDx) Drug: Placebo	Prometheus Biosciences, Inc.	Phase 2	Recruiting	No Results Available	
NCT05116969	DR3	anti-DR3 mAb	A Phase 1 Study of PTX-35 in Healthy Volunteers	Healthy Volunteer	Drug: PTX-35	Heat Biologics	Phase 1	Withdrawn	No Results Available	
NCT05107492	TL1A	anti-TL1A mAb	Evaluation of the Pharmacokinetics, Safety and Tolerability of Single Dose of PF-06480605 in Chinese Healthy Participants	Inflammatory Bowel Disease	Drug: 450mg Drug: 150mg Drug: Placebo	Pfizer	Phase 1	Completed	No Results Available	
NCT05013905	TL1A	anti-TL1A mAb	A Phase 2a Safety and Efficacy Open-Label Study of PRA023 in Subjects With Moderately to Severely Active Crohn’s Disease	Crohn Disease	Drug: PRA023 IV Device: Companion diagnostic (CDx)	Prometheus Biosciences, Inc.	Phase 2	Recruiting	No Results Available	
NCT04996797	TL1A	anti-TL1A mAb	A Phase 2 Safety and Efficacy Study of PRA023 in Subjects With Moderately to Severely Active Ulcerative Colitis	Ulcerative Colitis	Drug: PRA023 IV Device: Companion Diagnostic (CDx) Testing Other: Placebo	Prometheus Biosciences, Inc.	Phase 2	Recruiting	No Results Available	
NCT04676178	TL1A	anti-TL1A mAb	A Study of PRA023 in Healthy Volunteers	Healthy	Drug: PRA023 Other: Placebo	Prometheus Biosciences, Inc. Celerion	Phase 1	Completed	No Results Available	
NCT04269538	TL1A	anti-TL1A mAb	Evaluation of Safety, Tolerability and Pharmacokinetics of Single Dose of PF-06480605 in Japanese Healthy Participants	Healthy	Drug: PF-06480605 Drug: Placebo	Pfizer	Phase 1	Completed	No Results Available	
NCT04090411	TL1A	anti-TL1A mAb	A Study to Evaluate the Efficacy and Safety of PF-06480605 in Adult Participants With Moderate to Severe Ulcerative Colitis	Moderate to Severe Ulcerative Colitis	Drug: 50 mg Induction/Chronic Drug: 450 mg Induction/Chronic Drug: 150 mg Induction/Chronic Other: 0 mg Induction ONLY	Pfizer	Phase 2	Active, not recruiting	No Results Available	
NCT02840721	TL1A	anti-TL1A mAb	Safety, Efficacy, and Tolerability Study of PF-06480605 in Subjects With Moderate to Severe Ulcerative Colitis.	Colitis, Ulcerative	Drug: PF-06480605	Pfizer	Phase 2	Completed	Has Results	19-Jun-19
NCT01989143	TL1A	anti-TL1A mAb	Study to Evaluate the Safety, Tolerability, Pharmacokinetics and Pharmacodynamics of Single Intravenous and Multiple Subcutaneous and Intravenous Doses of PF-06480605 in Healthy Subjects.	Healthy	Drug: PF-06480605 Drug: Placebo	Pfizer	Phase 1	Completed	No Results Available	
NCT04905212	APRIL, BAFF	TACI-Fc fusion protein	A Study of Telitacicept for Injection (RC18) in Subjects With IgA Nephropathy	IgA Nephropathy	Drug: Telitacicept 160mg Drug: Telitacicept 240mg Drug: Placebo	RemeGen Co., Ltd.	Phase 2	Recruiting	No Results Available	
NCT04767698	BAFF	anti-BAFF mAb	Addition of Belimumab to B-cell Depletion in Relapsing-remitting Multiple Sclerosis	Multiple Sclerosis	Drug: Belimumab Drug: Short-course Ocrelizumab Drug: Continued Ocrelizumab	Johns Hopkins University GlaxoSmithKline	Phase 2	Terminated	No Results Available	
NCT04716231	APRIL, BAFF	TACI-Fc fusion protein	Atacicept in Subjects With IgA Nephropathy	IgA Nephropathy Berger Disease	Biological: Atacicept Other: Placebo to match Atacicept	Vera Therapeutics, Inc.	Phase 2	Active, not recruiting	No Results Available	
NCT04625153	APRIL, BAFF	TACI-Fc fusion protein	RC18 in Patients With Relapsing Remitting Multiple Sclerosis: Phase II Trial	Multiple Sclerosis, Relapsing-Remitting	Biological: RC18 160mg Biological: RC18 240mg	RemeGen Co., Ltd.	Phase 2	Recruiting	No Results Available	
NCT04291781	APRIL, BAFF	TACI-Fc fusion protein	A Study of RC18 Administered Subcutaneously to Subjects With IgA(Immunoglobulin A) Nephropathy	IgA Nephropathy	Biological: RC18 160mg Biological: RC18 240mg Biological: placebo	RemeGen Co., Ltd.	Phase 2	Completed	No Results Available	
NCT03844061	BAFF	anti-BAFF mAb	Belimumab and Rituximab Combination Therapy for the Treatment of Diffuse Cutaneous Systemic Sclerosis	Systemic Sclerosis	Drug: Belimumab Drug: Rituximab Other: Placebo Subcutaneous Injection Other: Placebo Infusion Drug: MMF	Hospital for Special Surgery, New York GlaxoSmithKline	Phase 2	Recruiting	No Results Available	
NCT03244059	BAFF	anti-BAFF mAb	Belimumab Treatment of Emphysema Patients With Anti-GRP78 Autoantibodies	Chronic Obstructive Pulmonary Disease Emphysema	Biological: Belimumab Drug: Placebo	University of Alabama at Birmingham GlaxoSmithKline	Phase 2	Completed	No Results Available	
NCT03016013	APRIL, BAFF	TACI-Fc fusion protein	A Study of the Efficacy and Safety of TACI-antibody Fusion Protein Injection (RC18) in Subjects With Inadequate Response to MTX Due to Treat Moderate and Severe Rheumatoid Arthritis.	Moderate and Severe RheumatoId Arthritis	Biological: Placebo plus MTX Biological: RC18 160 mg plus MTX	RemeGen Co., Ltd.	Phase 3	Active, not recruiting	No Results Available	
NCT02882087	APRIL, BAFF	TACI-Fc fusion protein	A Study of the Efficacy and Safety of TACI-antibody Fusion Protein Injection (RC18) in Subjects With Inadequate Response to TNF-𝛼 Antagonists Due to Treat Moderate and Severe Rheumatoid Arthritis	Moderate and Severe Rheumatoid Arthritis	Drug: Placebo plus MTX Drug: RC18 160 mg plus MTX	RemeGen Co., Ltd.	Phase 2	Terminated	No Results Available	
NCT02808429	APRIL, BAFF	TACI-Fc fusion protein	Efficacy and Safety of Atacicept in IgA Nephropathy	IgA Nephropathy	Drug: Placebo Drug: Atacicept 25 mg Drug: Atacicept 75 mg	EMD Serono Research & Development Institute, Inc. Merck KGaA, Darmstadt, Germany EMD Serono	Phase 2	Terminated	Has Results	25-Feb-21
NCT02062684	BAFF	anti-BAFF peptibody	BRIGHT-SC: Blisibimod Response in IgA Nephropathy Following At-Home Treatment by Subcutaneous Administration	IgA Nephropathy	Drug: Blisibimod Drug: Placebo	Anthera Pharmaceuticals	Phase 2|Phase 3	Completed	No Results Available	
NCT02052219	BAFF	anti-BAFF peptibody	BRILLIANT-SC: A Study of the Efficacy and Safety of Blisibimod Administration in Subjects With IgA Nephropathy	IgA Nephropathy	Drug: Blisibimod Drug: Placebo	Anthera Pharmaceuticals	Phase 3	Withdrawn	No Results Available	
NCT01676701	BAFF	anti-BAFF mAb	Evaluation of Tabalumab Using Auto-Injector or Prefilled Syringe in Participants With Rheumatoid Arthritis (RA)	Rheumatoid Arthritis	Drug: Tabalumab Auto-Injector Drug: Tabalumab Prefilled Syringe	Eli Lilly and Company	Phase 3	Terminated	Has Results	26-Apr-18
NCT01670565	BAFF	anti-BAFF mAb	Belimumab for the Treatment of Diffuse Cutaneous Systemic Sclerosis	Systemic Sclerosis	Drug: Belimumab Drug: Mycophenolate Mofetil Other: Placebo Infusion	Hospital for Special Surgery, New York Human Genome Sciences Inc.	Phase 2	Completed	Has Results	24-May-22
NCT01576549	BAFF	anti-BAFF mAb	A Study of LY2127399 in Rheumatoid Arthritis	Rheumatoid Arthritis	Drug: LY2127399	Eli Lilly and Company	Phase 2	Terminated	Has Results	26-Apr-18
NCT01253291	BAFF	anti-BAFF mAb	A Study of Japanese Rheumatoid Arthritis Participants	Rheumatoid Arthritis	Drug: LY2127399	Eli Lilly and Company	Phase 1	Completed	Has Results	1-Mar-19
NCT01253226	BAFF	anti-BAFF mAb	A Study for Japanese Participants With Rheumatoid Arthritis (RA)	Rheumatoid Arthritis	Drug: LY2127399 (Tabalumab) Drug: Placebo	Eli Lilly and Company	Phase 1	Completed	Has Results	23-Oct-18
NCT01215942	BAFF	anti-BAFF mAb	An Open Label Study for Participants With Rheumatoid Arthritis	Rheumatoid Arthritis	Drug: LY2127399	Eli Lilly and Company	Phase 3	Terminated	Has Results	11-Jun-18
NCT01202773	BAFF	anti-BAFF mAb	A Study in Participants With Rheumatoid Arthritis	Rheumatoid Arthritis	Drug: LY2127399 Drug: Placebo Q4W Drug: Placebo Q2W	Eli Lilly and Company	Phase 3	Terminated	Has Results	14-May-18
NCT01202760	BAFF	anti-BAFF mAb	A Rheumatoid Arthritis Study in Participants	Rheumatoid Arthritis	Drug: LY2127399 Drug: Placebo	Eli Lilly and Company	Phase 3	Completed	Has Results	25-Apr-18
NCT01198002	BAFF	anti-BAFF mAb	A Rheumatoid Arthritis Study in Participants on a Background Treatment of Methotrexate	Rheumatoid Arthritis	Drug: LY2127399 Drug: Placebo Q2W Drug: Placebo Q4W Drug: Methotrexate	Eli Lilly and Company	Phase 3	Terminated	Has Results	8-May-18
NCT00931086	BAFF	anti-BAFF mAb	Expanded Access Trial of Belimumab Antibody in RA Patients Who Were Previously Treated Under HGS Protocol LBRA99	Rheumatoid Arthritis	Drug: belimumab	Human Genome Sciences Inc., a GSK Company GlaxoSmithKline		No longer available	No Results Available	
NCT00882999	BAFF	anti-BAFF mAb	A Study of Participants With Relapsing-Remitting Multiple Sclerosis (RRMS)	Relapsing-Remitting Multiple Sclerosis	Drug: LY2127399 Drug: Placebo	Eli Lilly and Company	Phase 2	Completed	Has Results	15-Nov-18
NCT00853762	APRIL, BAFF	TACI-Fc fusion protein	Atacicept in Multiple Sclerosis Extension Study, Phase II	Relapsing Multiple Sclerosis	Drug: Atacicept 25 mg Drug: Atacicept 75 mg Drug: Atacicept 150 mg	EMD Serono Merck KGaA, Darmstadt, Germany	Phase 2	Terminated	Has Results	24-May-16
NCT00837811	BAFF	anti-BAFF mAb	An Open Label Extension Study in Participants With Rheumatoid Arthritis	Rheumatoid Arthritis	Biological: LY2127399	Eli Lilly and Company	Phase 2	Completed	Has Results	25-Apr-18
NCT00785928	BAFF	anti-BAFF mAb	A Study for Patients With Active Rheumatoid Arthritis Despite Ongoing Methotrexate Therapy	Rheumatoid Arthritis	Biological: LY2127399 Drug: Placebo	Eli Lilly and Company	Phase 2	Completed	Has Results	10-Jul-18
NCT00689728	BAFF	anti-BAFF mAb	A Study for Patients With Rheumatoid Arthritis on Methotrexate (MTX) With an Inadequate Response to TNF𝛼 Inhibitor Therapy	Arthritis, Rheumatoid	Biological: LY2127399 Drug: Placebo	Eli Lilly and Company	Phase 2	Completed	Has Results	6-Dec-18
NCT00664521	APRIL, BAFF	TACI-Fc fusion protein	Atacicept in Combination With Rituximab in Subjects With Rheumatoid Arthritis (August III)	Rheumatoid Arthritis	Biological: Rituximab Drug: Atacicept Drug: Placebo matched to atacicept	Merck KGaA, Darmstadt, Germany	Phase 2	Completed	Has Results	30-Dec-16
NCT00642902	APRIL, BAFF	TACI-Fc fusion protein	A Phase 2 Study of Atacicept in Subjects With Relapsing Multiple Sclerosis (ATAMS)	Relapsing Multiple Sclerosis	Drug: Atacicept Drug: Placebo matched to atacicept	EMD Serono	Phase 2	Terminated	Has Results	24-May-16
NCT00595413	APRIL, BAFF	TACI-Fc fusion protein	Atacicept in Anti-Tumor Necrosis Factor Alpha-naÏve Subjects With Rheumatoid Arthritis (AUGUST II)	Rheumatoid Arthritis	Drug: Placebo matched to atacicept Drug: Atacicept: with loading dose Drug: Atacicept Biological: Adalimumab	EMD Serono Merck KGaA, Darmstadt, Germany	Phase 2	Completed	Has Results	17-Feb-16
NCT00583557	BAFF	anti-BAFF mAb	A Continuation Trial for Subjects With Rheumatoid Arthritis That Have Completed Protocol LBRA01	Rheumatoid Arthritis	Drug: belimumab	Human Genome Sciences Inc.	Phase 2	Terminated	Has Results	29-Jun-11
NCT00430495	APRIL, BAFF	TACI-Fc fusion protein	A Phase 2 Dose-finding Study of Atacicept in Subjects With Rheumatoid Arthritis (AUGUST I)	Rheumatoid Arthritis	Drug: Atacicept Drug: Placebo matched to atacicept	EMD Serono Merck KGaA, Darmstadt, Germany	Phase 2	Completed	Has Results	17-Feb-16
NCT00308282	BAFF	anti-BAFF mAb	A Multi-Site Study to Evaluate the Safety and Effect of Study Drug on Participants With Rheumatoid Arthritis	Arthritis, Rheumatoid	Drug: LY2127399 Drug: Placebo	Eli Lilly and Company	Phase 2	Completed	Has Results	18-Mar-19
NCT00071812	BAFF	anti-BAFF mAb	A Safety and Efficacy Study of LymphoStat-B™ (Monoclonal Anti-BLyS Antibody) in Subjects With Rheumatoid Arthritis (RA)	Arthritis, Rheumatoid	Drug: Placebo Drug: Belimumab 1 mg/kg Drug: Belimumab 4 mg/kg Drug: Belimumab 10 mg/kg	Human Genome Sciences Inc.	Phase 2	Completed	Has Results	25-Jun-12
NCT02321280	RANKL	anti-RANKL mAb	The Efficacy of Denosumab in Active Crohn’s Disease	Crohn Disease	Drug: Denosumab	University of Manitoba University of Toronto McMaster University	Phase 1 Phase 2	Completed	No Results Available	
NCT02132026	RANKL	anti-RANKL mAb	Study Investigating the Effect of Drugs Used to Treat Osteoporosis on the Progression of Calcific Aortic Stenosis.	Calcific Aortic Stenosis	Drug: Denosumab Drug: Alendronic Acid Drug: Denosumab Placebo Drug: Alendronic Acid Placebo	University of Edinburgh British Heart Foundation NHS Lothian	Phase 2	Completed	No Results Available	
NCT00983658	OX40L	anti-OX40L mAb	A Study of huMAb OX40L in the Prevention of Allergen-Induced Airway Obstruction in Adults With Mild Allergic Asthma	Asthma	Drug: huMAb OX40L Drug: placebo	Genentech, Inc.	Phase 2	Completed	No Results Available	
NCT02647866	OX40	anti-OX40 mAb	Study of a Monoclonal Antibody KHK4083 in Moderate Ulcerative Colitis	Ulcerative Colitis Digestive System Diseases Colitis, Ulcerative Colitis Gastrointestinal Diseases Inflammatory Bowel Diseases Intestinal Diseases Colonic Diseases Autoimmune Disease Abdominal Pain	Drug: KHK4083 Drug: Placebo	Kyowa Kirin, Inc.	Phase 2	Completed	Has Results	5-Mar-20
NCT03161288	OX40L	anti-OX40L mAb	A Study of KY1005 in Healthy Volunteers	Immune System Diseases	Drug: KY1005 Drug: Placebo	Kymab Limited	Phase 1	Completed	No Results Available	
NCT03568162	OX40L	anti-OX40L mAb	Phase 2b Study to Evaluate the Efficacy and Safety of ISB 830 in Adults With Moderate to Severe Atopic Dermatitis	Moderate to Severe Atopic Dermatitis	Drug: ISB 830 - Part 1 Group 1 Drug: ISB 830 - Part 1 Group 2 Drug: ISB 830 - Part 1 Group 3 Drug: Placebo - Part 1 Group 4 Drug: ISB 830 - Part 2 Group 5 Drug: Placebo - Part 2 Group 6	Ichnos Sciences SA Glenmark Pharmaceuticals S.A.	Phase 2	Active, not recruiting	Has Results	28-Jun-22
NCT03703102	OX40	anti-OX40 mAb	Study of an Anti-OX40 Monoclonal Antibody (KHK4083) in Subjects With Moderate to Severe Atopic Dermatitis	Atopic dermatitis	Drug: KHK4083 Drug: Placebo	Kyowa Kirin, Inc. Kyowa Kirin Co., Ltd.	Phase 2	Completed	Has Results	9-Jun-22
NCT03754309	OX40L	anti-OX40L mAb	A Study of KY1005 in Patients With Moderate to Severe Atopic Dermatitis	Dermatitis, atopic	Drug: KY1005 Drug: Placebo	Kymab Limited	Phase 2	Completed	No Results Available	
NCT04449939	OX40L	anti-OX40L mAb	A Study of Subcutaneous KY1005 in Healthy Volunteers	Immune System Diseases	Drug: KY1005	Kymab Limited	Phase 1	Completed	No Results Available	

Clinical trials in Phases 1-3 targeting TNFSF members or their receptors to treat diseases presenting with fibrosis. Due to the successful results and FDA approval of anti-TNFα therapies, please note that all clinical trials targeting TNFα could not be listed due to space constraints. These additional trials can be found at https://clinicaltrials.gov/.

## Author contributions

HS, RH, VT, JC, AW, GD, JB, EC, and AG all contributed to writing this manuscript. HS made the Figures and Tables. HS and RH edited the manuscript. All authors contributed to the article and approved the submitted version.

## References

[1] Wynn TA. Fibrotic disease and the T(H)1/T(H)2 paradigm. Nat Rev Immunol (2004) 4:583–94. doi: 10.1038/nri1412 PMC2702150 15286725

[2] Herro R Da Silva Antunes R Aguilera AR Tamada K Croft M. Tumor necrosis factor superfamily 14 (LIGHT) controls thymic stromal lymphopoietin to drive pulmonary fibrosis. J Allergy Clin Immunol (2015) 136:757–68. doi: 10.1016/j.jaci.2014.12.1936 PMC4532661 25680454

[3] Herro R Antunes RDS Aguilera AR Tamada K Croft M. The tumor necrosis factor superfamily molecule LIGHT promotes keratinocyte activity and skin fibrosis. J Invest Dermatol (2015) 135:2109–18. doi: 10.1038/jid.2015.110 PMC4504809 25789702

[4] Herro R Shui JW Zahner S Sidler D Kawakami Y Kawakami T. LIGHT-HVEM signaling in keratinocytes controls development of dermatitis. J Exp Med (2018) 215:415–22. doi: 10.1084/jem.20170536 PMC5789407 29339444

[5] Herro R Miki H Sethi GS Mills D Mehta AK Nguyen XX. TL1A promotes lung tissue fibrosis and airway remodeling. J Immunol (2020) 205:2414–22. doi: 10.4049/jimmunol.2000665 PMC7577982 32958689

[6] Carswell EA Old LJ Kassel RL Green S Fiore N Williamson B. An endotoxin-induced serum factor that causes necrosis of tumors. Proc Natl Acad Sci U S A (1975) 72:3666–70. doi: 10.1073/pnas.72.9.3666 PMC433057 1103152

[7] Aggarwal BB Moffat B Harkins RN. Human lymphotoxin. production by a lymphoblastoid cell line, purification, and initial characterization. J Biol Chem (1984) 259:686–91. doi: 10.1016/S0021-9258(17)43716-1 6608523

[8] Black RA Rauch CT Kozlosky CJ Peschon JJ Slack JL Wolfson MF. A metalloproteinase disintegrin that releases tumour-necrosis factor-alpha from cells. Nature (1997) 385:729–33. doi: 10.1038/385729a0 9034190

[9] Grivennikov SI Tumanov AV Liepinsh DJ Kruglov AA Marakusha BI Shakhov AN. Distinct and nonredundant *in vivo* functions of TNF produced by t cells and macrophages/neutrophils: protective and deleterious effects. Immunity (2005) 22:93–104. doi: 10.1016/j.immuni.2004.11.016 15664162

[10] Loetscher H Steinmetz M Lesslauer W. Tumor necrosis factor: receptors and inhibitors. Cancer Cells (1991) 3:221–6. 1654969

[11] Carpentier I Coornaert B Beyaert R. Function and regulation of tumor necrosis factor receptor type 2. Curr Med Chem (2004) 11:2205–12. doi: 10.2174/0929867043364694 15279559

[12] Grell M Douni E Wajant H Lohden M Clauss M Maxeiner B. The transmembrane form of tumor necrosis factor is the prime activating ligand of the 80 kDa tumor necrosis factor receptor. Cell (1995) 83:793–802. doi: 10.1016/0092-8674(95)90192-2 8521496

[13] Boschert V Krippner-Heidenreich A Branschadel M Tepperink J Aird A Scheurich P. Single chain TNF derivatives with individually mutated receptor binding sites reveal differential stoichiometry of ligand receptor complex formation for TNFR1 and TNFR2. Cell Signal (2010) 22:1088–96. doi: 10.1016/j.cellsig.2010.02.011 20206684

[14] Richter C Messerschmidt S Holeiter G Tepperink J Osswald S Zappe A. The tumor necrosis factor receptor stalk regions define responsiveness to soluble versus membrane-bound ligand. Mol Cell Biol (2012) 32:2515–29. doi: 10.1128/MCB.06458-11 PMC3434479 22547679

[15] Hsu H Xiong J Goeddel DV. The TNF receptor 1-associated protein TRADD signals cell death and NF-kappa b activation. Cell (1995) 81:495–504. doi: 10.1016/0092-8674(95)90070-5 7758105

[16] Rothe M Sarma V Dixit VM Goeddel DV. TRAF2-mediated activation of NF-kappa b by TNF receptor 2 and CD40. Science (1995) 269:1424–7. doi: 10.1126/science.7544915 7544915

[17] Heilig B Fiehn C Brockhaus M Gallati H Pezzutto A Hunstein W. Evaluation of soluble tumor necrosis factor (TNF) receptors and TNF receptor antibodies in patients with systemic lupus erythematodes, progressive systemic sclerosis, and mixed connective tissue disease. J Clin Immunol (1993) 13:321–8. doi: 10.1007/BF00920240 8245178

[18] Majewski S Wojas-Pelc A Malejczyk M Szymanska E Jablonska S. Serum levels of soluble TNF alpha receptor type I and the severity of systemic sclerosis. Acta Derm Venereol (1999) 79:207–10. doi: 10.1080/000155599750010986 10384918

[19] Terao M Murota H Kitaba S Katayama I. Tumor necrosis factor-alpha processing inhibitor-1 inhibits skin fibrosis in a bleomycin-induced murine model of scleroderma. Exp Dermatol (2010) 19:38–43. doi: 10.1111/j.1600-0625.2009.00973.x 19758314

[20] Hugle T O'Reilly S Simpson R Kraaij MD Bigley V Collin M. Tumor necrosis factor-costimulated T lymphocytes from patients with systemic sclerosis trigger collagen production in fibroblasts. Arthritis Rheum (2013) 65:481–91. doi: 10.1002/art.37738 PMC6588536 23045159

[21] Chavez-Galan L Becerril C Ruiz A Ramon-Luing LA Cisneros J Montano M. Fibroblasts from idiopathic pulmonary fibrosis induce apoptosis and reduce the migration capacity of T lymphocytes. Front Immunol (2022) 13:820347. doi: 10.3389/fimmu.2022.820347 35222396 PMC8866565

[22] Piguet PF Collart MA Grau GE Kapanci Y Vassalli P. Tumor necrosis factor/cachectin plays a key role in bleomycin-induced pneumopathy and fibrosis. J Exp Med (1989) 170:655–63. doi: 10.1084/jem.170.3.655 PMC2189418 2475571

[23] Oikonomou N Harokopos V Zalevsky J Valavanis C Kotanidou A Szymkowski DE. Soluble TNF mediates the transition from pulmonary inflammation to fibrosis. PLoS One (2006) 1:e108. doi: 10.1371/journal.pone.0000108 17205112 PMC1762410

[24] Redente EF Keith RC Janssen W Henson PM Ortiz LA Downey GP. Tumor necrosis factor-alpha accelerates the resolution of established pulmonary fibrosis in mice by targeting profibrotic lung macrophages. Am J Respir Cell Mol Biol (2014) 50:825–37. doi: 10.1165/rcmb.2013-0386OC PMC4068926 24325577

[25] Li XM Chen X Gu W Guo YJ Cheng Y Peng J. Impaired TNF/TNFR2 signaling enhances Th2 and Th17 polarization and aggravates allergic airway inflammation. Am J Physiol Lung Cell Mol Physiol (2017) 313:L592–601. doi: 10.1152/ajplung.00409.2016 28619762

[26] Chavez-Galan L Buendia-Roldan I Castillo-Castillo K Preciado-Garcia M Ocana-Guzman R Salgado A. Decreased expression of transmembrane TNFR2 in lung leukocytes subpopulations of patients with non-fibrotic hypersensitivity pneumonitis compared with the fibrotic disease. Clin Immunol (2020) 215:108424. doi: 10.1016/j.clim.2020.108424 32305453

[27] Goldberg MT Han YP Yan C Shaw MC Garner WL. TNF-alpha suppresses alpha-smooth muscle actin expression in human dermal fibroblasts: an implication for abnormal wound healing. J Invest Dermatol (2007) 127:2645–55. doi: 10.1038/sj.jid.5700890 PMC2366884 17554369

[28] Verjee LS Verhoekx JS Chan JK Krausgruber T Nicolaidou V Izadi D. Unraveling the signaling pathways promoting fibrosis in dupuytren's disease reveals TNF as a therapeutic target. Proc Natl Acad Sci U S A (2013) 110:E928–37. doi: 10.1073/pnas.1301100110 PMC3593900 23431165

[29] Izadi D Layton TB Williams L McCann F Cabrita M Espirito Santo AI. Identification of TNFR2 and IL-33 as therapeutic targets in localized fibrosis. Sci Adv (2019) 5:eaay0370. doi: 10.1126/sciadv.aay0370 31840071 PMC6892635

[30] Theiss AL Simmons JG Jobin C Lund PK. Tumor necrosis factor (TNF) alpha increases collagen accumulation and proliferation in intestinal myofibroblasts via TNF receptor 2. J Biol Chem (2005) 280:36099–109. doi: 10.1074/jbc.M505291200 16141211

[31] Sudo K Yamada Y Moriwaki H Saito K Seishima M. Lack of tumor necrosis factor receptor type 1 inhibits liver fibrosis induced by carbon tetrachloride in mice. Cytokine (2005) 29:236–44. doi: 10.1016/j.cyto.2004.11.001 15760680

[32] Abdul-Hamid M Ahmed RR Moustafa N Nady R. The antifibrogenic effect of etanercept on development of liver cirrhosis induced by thioacetamide in rats. Ultrastruct Pathol (2017) 41:23–35. doi: 10.1080/01913123.2016.1256361 27982723

[33] Wandrer F Liebig S Marhenke S Vogel A John K Manns MP. TNF-Receptor-1 inhibition reduces liver steatosis, hepatocellular injury and fibrosis in NAFLD mice. Cell Death Dis (2020) 11:212. doi: 10.1038/s41419-020-2411-6 32235829 PMC7109108

[34] Benoot T Piccioni E De Ridder K Goyvaerts C. TNFalpha and immune checkpoint inhibition: friend or foe for lung cancer? Int J Mol Sci (2021) 17:8691. doi: 10.3390/ijms22168691 PMC8395431 34445397

[35] Nicolaou A Zhao Z Northoff BH Sass K Herbst A Kohlmaier A. Adam17 deficiency promotes atherosclerosis by enhanced TNFR2 signaling in mice. Arterioscler Thromb Vasc Biol (2017) 37:247–57. doi: 10.1161/ATVBAHA.116.308682 28062509

[36] Sacca R Cuff CA Lesslauer W Ruddle NH. Differential activities of secreted lymphotoxin-alpha3 and membrane lymphotoxin-alpha1beta2 in lymphotoxin-induced inflammation: critical role of TNF receptor 1 signaling. J Immunol (1998) 160:485–91. doi: 10.4049/jimmunol.160.1.485 9552007

[37] Duerrschmid C Crawford JR Reineke E Taffet GE Trial J Entman ML. TNF receptor 1 signaling is critically involved in mediating angiotensin-II-induced cardiac fibrosis. J Mol Cell Cardiol (2013) 57:59–67. doi: 10.1016/j.yjmcc.2013.01.006 23337087 PMC3593947

[38] Duerrschmid C Trial J Wang Y Entman ML Haudek SB. Tumor necrosis factor: a mechanistic link between angiotensin-II-induced cardiac inflammation and fibrosis. Circ Heart Fail (2015) 8:352–61. doi: 10.1161/CIRCHEARTFAILURE.114.001893 PMC4366299 25550440

[39] Miao K Zhou L Ba H Li C Gu H Yin B. 'Transmembrane tumor necrosis factor alpha attenuates pressure-overload cardiac hypertrophy via tumor necrosis factor receptor 2'. PLoS Biol (2020) 18:e3000967. doi: 10.1371/journal.pbio.3000967 33270628 PMC7714153

[40] Besse S Nadaud S Balse E Pavoine C. Early protective role of inflammation in cardiac remodeling and heart failure: focus on TNFalpha and resident macrophages. Cells (2022) 11:1249. doi: 10.3390/cells11071249 35406812 PMC8998130

[41] Gonzalez A Schelbert EB Diez J Butler J. Myocardial interstitial fibrosis in heart failure: biological and translational perspectives. J Am Coll Cardiol (2018) 71:1696–706. doi: 10.1016/j.jacc.2018.02.021 29650126

[42] Sweeney M Corden B Cook SA. Targeting cardiac fibrosis in heart failure with preserved ejection fraction: mirage or miracle? EMBO Mol Med (2020) 12:e10865. doi: 10.15252/emmm.201910865 32955172 PMC7539225

[43] Monden Y Kubota T Inoue T Tsutsumi T Kawano S Ide T. 'Tumor necrosis factor-alpha is toxic via receptor 1 and protective via receptor 2 in a murine model of myocardial infarction'. Am J Physiol Heart Circ Physiol (2007) 293:H743–53. doi: 10.1152/ajpheart.00166.2007 17416608

[44] Zhang Y Zhao J Lau WB Jiao LY Liu B Yuan Y. Tumor necrosis factor-alpha and lymphotoxin-alpha mediate myocardial ischemic injury via TNF receptor 1, but are cardioprotective when activating TNF receptor 2. PLoS One (2013) 8:e60227. doi: 10.1371/journal.pone.0060227 23704873 PMC3660398

[45] Mauri DN Ebner R Montgomery RI Kochel KD Cheung TC Yu GL. 'LIGHT, a new member of the TNF superfamily, and lymphotoxin alpha are ligands for herpesvirus entry mediator'. Immunity (1998) 8:21–30. doi: 10.1016/S1074-7613(00)80455-0 9462508

[46] Yu KY Kwon B Ni J Zhai Y Ebner R Kwon BS. A newly identified member of tumor necrosis factor receptor superfamily (TR6) suppresses LIGHT-mediated apoptosis. J Biol Chem (1999) 274:13733–6. doi: 10.1074/jbc.274.20.13733 10318773

[47] Harrop JA McDonnell PC Brigham-Burke M Lyn SD Minton J Tan KB. Herpesvirus entry mediator ligand (HVEM-l), a novel ligand for HVEM/TR2, stimulates proliferation of T cells and inhibits HT29 cell growth. J Biol Chem (1998) 273:27548–56. doi: 10.1074/jbc.273.42.27548 9765287

[48] Schneider K Potter KG Ware CF. 'Lymphotoxin and LIGHT signaling pathways and target genes'. Immunol Rev (2004) 202:49–66. doi: 10.1111/j.0105-2896.2004.00206.x 15546385

[49] Crowe PD VanArsdale TL Walter BN Ware CF Hession C Ehrenfels B. A lymphotoxin-beta-specific receptor. Science (1994) 264:707–10. doi: 10.1126/science.8171323 8171323

[50] Shou Y Koroleva E Spencer CM Shein SA Korchagina AA Yusoof KA. Redefining the role of lymphotoxin beta receptor in the maintenance of lymphoid organs and immune cell homeostasis in adulthood. Front Immunol (2021) 12:712632. doi: 10.3389/fimmu.2021.712632 34335629 PMC8320848

[51] Wynn TA Ramalingam TR. Mechanisms of fibrosis: therapeutic translation for fibrotic disease. Nat Med (2012) 18:1028–40. doi: 10.1038/nm.2807 PMC3405917 22772564

[52] Doherty TA Soroosh P Khorram N Fukuyama S Rosenthal P Cho JY. The tumor necrosis factor family member LIGHT is a target for asthmatic airway remodeling. Nat Med (2011) 17:596–603. doi: 10.1038/nm.2356 21499267 PMC3097134

[53] Qu HQ Snyder J Connolly J Glessner J Kao C Sleiman PMA. Circulating LIGHT (TNFSF14) and interleukin-18 levels in sepsis-induced multi-organ injuries. medRxiv (2022) 10(2):264–76. doi: 10.1101/2021.05.25.21257799 PMC8869623 35203474

[54] Masuoka M Shiraishi H Ohta S Suzuki S Arima K Aoki S. Periostin promotes chronic allergic inflammation in response to Th2 cytokines. J Clin Invest (2012) 122:2590–600. doi: 10.1172/JCI58978 PMC3386810 22684102

[55] Ikawa T Ichimura Y Miyagawa T Fukui Y Toyama S Omatsu J. The contribution of LIGHT (TNFSF14) to the development of systemic sclerosis by modulating IL-6 and T helper type 1 chemokine expression in dermal fibroblasts. J Invest Dermatol (2022) 142:1541–51.e3. doi: 10.1016/j.jid.2021.10.028 34838790

[56] Otterdal K Haukeland JW Yndestad A Dahl TB Holm S Segers FM. Increased serum levels of LIGHT/TNFSF14 in nonalcoholic fatty liver disease: possible role in hepatic inflammation. Clin Transl Gastroenterol (2015) 6:e95. doi: 10.1038/ctg.2015.23 26133108 PMC4816254

[57] Herrero-Cervera A Vinue A Burks DJ Gonzalez-Navarro H. Genetic inactivation of the LIGHT (TNFSF14) cytokine in mice restores glucose homeostasis and diminishes hepatic steatosis. Diabetologia (2019) 62:2143–57. doi: 10.1007/s00125-019-4962-6 31388695

[58] Liang QS Xie JG Yu C Feng Z Ma J Zhang Y. Splenectomy improves liver fibrosis via tumor necrosis factor superfamily 14 (LIGHT) through the JNK/TGF-beta1 signaling pathway. Exp Mol Med (2021) 53:393–406. doi: 10.1038/s12276-021-00574-2 33654222 PMC8080781

[59] Manresa MC Chiang AWT Kurten RC Dohil R Brickner H Dohil L. Increased production of LIGHT by T cells in eosinophilic esophagitis promotes differentiation of esophageal fibroblasts toward an inflammatory phenotype. Gastroenterology (2020) 159:1778–92.e13. doi: 10.1053/j.gastro.2020.07.035 32712105 PMC7726704

[60] Li Y Tang M Han B Wu S Li SJ He QH. Tumor necrosis factor superfamily 14 is critical for the development of renal fibrosis. Aging (Albany NY) (2020) 12:25469–86. doi: 10.18632/aging.104151 PMC7803499 33231567

[61] Bielecki P Gindzienska-Sieskiewicz E Reszec J Piszczatowski B Rogowski M Kowal-Bielecka O. Expression of LIGHT/TNFSF14 and its receptors, HVEM and LTbetaR, correlates with the severity of fibrosis in lacrimal sacs from patients with lacrimal duct obstruction. Ophthalmol Ther (2021) 10:63–74. doi: 10.1007/s40123-020-00320-3 33188486 PMC7665092

[62] Migone TS Zhang J Luo X Zhuang L Chen C Hu B. 'TL1A is a TNF-like ligand for DR3 and TR6/DcR3 and functions as a T cell costimulator'. Immunity (2002) 16:479–92. doi: 10.1016/S1074-7613(02)00283-2 11911831

[63] Bamias G Martin C Marini M Hoang S Mishina M Ross WG. Expression, localization, and functional activity of TL1A, a novel Th1-polarizing cytokine in inflammatory bowel disease. J Immunol (2003) 171:4868–74. doi: 10.4049/jimmunol.171.9.4868 14568967

[64] Meylan F Hawley ET Barron L Barlow JL Penumetcha P Pelletier M. 'The TNF-family cytokine TL1A promotes allergic immunopathology through group 2 innate lymphoid cells'. Mucosal Immunol (2014) 7:958–68. doi: 10.1038/mi.2013.114 PMC4165592 24368564

[65] Pitti RM Marsters SA Lawrence DA Roy M Kischkel FC Dowd P. Genomic amplification of a decoy receptor for fas ligand in lung and colon cancer. Nature (1998) 396:699–703. doi: 10.1038/25387 9872321

[66] Yu X Pappu R Ramirez-Carrozzi V Ota N Caplazi P Zhang J. TNF superfamily member TL1A elicits type 2 innate lymphoid cells at mucosal barriers. Mucosal Immunol (2014) 7:730–40. doi: 10.1038/mi.2013.92 PMC3998636 24220298

[67] Clarke AW Poulton L Shim D Mabon D Butt D Pollard M. An anti-TL1A antibody for the treatment of asthma and inflammatory bowel disease. MAbs (2018) 10:664–77. doi: 10.1080/19420862.2018.1440164 PMC5973687 29436901

[68] Ferdinand JR Richard AC Meylan F Al-Shamkhani A Siegel RM. Cleavage of TL1A differentially regulates its effects on innate and adaptive immune cells. J Immunol (2018) 200:1360–69. doi: 10.4049/jimmunol.1700891 PMC5812441 29335258

[69] Meylan F Song YJ Fuss I Villarreal S Kahle E Malm IJ. 'The TNF-family cytokine TL1A drives IL-13-dependent small intestinal inflammation'. Mucosal Immunol (2011) 4:172–85. doi: 10.1038/mi.2010.67 PMC3437258 20980995

[70] Jacob N Kumagai K Abraham JP Shimodaira Y Ye Y Luu J. Direct signaling of TL1A-DR3 on fibroblasts induces intestinal fibrosis *in vivo* . Sci Rep (2020) 10:18189. doi: 10.1038/s41598-020-75168-5 33097818 PMC7584589

[71] Wenxiu J Mingyue Y Fei H Yuxin L Mengyao W Chenyang L. Effect and mechanism of TL1A expression on epithelial-mesenchymal transition during chronic colitis-related intestinal fibrosis. Mediators Inflammation (2021) 2021:5927064. doi: 10.1155/2021/5927064 PMC8253633 34257516

[72] Hassan-Zahraee M Ye Z Xi L Baniecki ML Li X Hyde CL. Antitumor necrosis factor-like ligand 1A therapy targets tissue inflammation and fibrosis pathways and reduces gut pathobionts in ulcerative colitis. Inflammation Bowel Dis (2022) 28:434–46. doi: 10.1093/ibd/izab193 PMC8889296 34427649

[73] Shih DQ Zheng L Zhang X Zhang H Kanazawa Y Ichikawa R. Inhibition of a novel fibrogenic factor Tl1a reverses established colonic fibrosis. Mucosal Immunol (2014) 7:1492–503. doi: 10.1038/mi.2014.37 PMC4205266 24850426

[74] Zhang J Zhang D Pan Y Liu X Xu J Qiao X. The TL1A-DR3 axis in asthma: membrane-bound and secreted TL1A Co-determined the development of airway remodeling. Allergy Asthma Immunol Res (2022) 14:233–53. doi: 10.4168/aair.2022.14.2.233 PMC8914606 35255540

[75] Steele H Sachen K McKnight AJ Soloff R Herro R. Targeting TL1A/DR3 signaling offers a therapeutic advantage to neutralizing IL13/IL4Ralpha in muco-secretory fibrotic disorders. Front Immunol (2021) 12:692127. doi: 10.3389/fimmu.2021.692127 34305924 PMC8299868

[76] Guo J Luo Y Yin F Huo X Niu G Song M. Overexpression of tumor necrosis factor-like ligand 1 a in myeloid cells aggravates liver fibrosis in mice. J Immunol Res (2019) 2019:7657294. doi: 10.1155/2019/7657294 30906791 PMC6393882

[77] Hahne M Kataoka T Schroter M Hofmann K Irmler M Bodmer JL. APRIL, a new ligand of the tumor necrosis factor family, stimulates tumor cell growth. J Exp Med (1998) 188:1185–90. doi: 10.1084/jem.188.6.1185 PMC2212534 9743536

[78] Shu HB Hu WH Johnson H. TALL-1 is a novel member of the TNF family that is down-regulated by mitogens. J Leukoc Biol (1999) 65:680–3. doi: 10.1002/jlb.65.5.680 10331498

[79] Moore PA Belvedere O Orr A Pieri K LaFleur DW Feng P. 'BLyS: member of the tumor necrosis factor family and b lymphocyte stimulator'. Science (1999) 285:260–3. doi: 10.1126/science.285.5425.260 10398604

[80] Schneider P MacKay F Steiner V Hofmann K Bodmer JL Holler N. BAFF, a novel ligand of the tumor necrosis factor family, stimulates b cell growth. J Exp Med (1999) 189:1747–56. doi: 10.1084/jem.189.11.1747 PMC2193079 10359578

[81] Lopez-Fraga M Fernandez R Albar JP Hahne M. Biologically active APRIL is secreted following intracellular processing in the golgi apparatus by furin convertase. EMBO Rep (2001) 2:945–51. doi: 10.1093/embo-reports/kve198 PMC1084076 11571266

[82] Nardelli B Belvedere O Roschke V Moore PA Olsen HS Migone TS. Synthesis and release of b-lymphocyte stimulator from myeloid cells. Blood (2001) 97:198–204. doi: 10.1182/blood.V97.1.198 11133761

[83] Gras MP Laabi Y Linares-Cruz G Blondel MO Rigaut JP Brouet JC. BCMAp: an integral membrane protein in the golgi apparatus of human mature b lymphocytes. Int Immunol (1995) 7:1093–106. doi: 10.1093/intimm/7.7.1093 8527407

[84] von Bulow GU Bram RJ. NF-AT activation induced by a CAML-interacting member of the tumor necrosis factor receptor superfamily. Science (1997) 278:138–41. doi: 10.1126/science.278.5335.138 9311921

[85] Lentz VM Hayes CE Cancro MP. Bcmd decreases the life span of b-2 but not b-1 cells in A/WySnJ mice. J Immunol (1998) 160:3743–7. doi: 10.4049/jimmunol.160.8.3743 9558076

[86] Madry C Laabi Y Callebaut I Roussel J Hatzoglou A Le Coniat M. The characterization of murine BCMA gene defines it as a new member of the tumor necrosis factor receptor superfamily. Int Immunol (1998) 10:1693–702. doi: 10.1093/intimm/10.11.1693 9846698

[87] Thompson JS Bixler SA Qian F Vora K Scott ML Cachero TG. BAFF-r, a newly identified TNF receptor that specifically interacts with BAFF. Science (2001) 293:2108–11. doi: 10.1126/science.1061965 11509692

[88] Yan M Brady JR Chan B Lee WP Hsu B Harless S. Identification of a novel receptor for b lymphocyte stimulator that is mutated in a mouse strain with severe b cell deficiency. Curr Biol (2001) 11:1547–52. doi: 10.1016/S0960-9822(01)00481-X 11591325

[89] Bossen C Schneider P. BAFF, APRIL and their receptors: structure, function and signaling. Semin Immunol (2006) 18:263–75. doi: 10.1016/j.smim.2006.04.006 16914324

[90] Kampa M Notas G Stathopoulos EN Tsapis A Castanas E. The TNFSF members APRIL and BAFF and their receptors TACI, BCMA, and BAFFR in oncology, with a special focus in breast cancer. Front Oncol (2020) 10:827. doi: 10.3389/fonc.2020.00827 32612943 PMC7308424

[91] Allman WR Dey R Liu L Siddiqui S Coleman AS Bhattacharya P. TACI deficiency leads to alternatively activated macrophage phenotype and susceptibility to leishmania infection. Proc Natl Acad Sci U S A (2015) 112:E4094–103. doi: 10.1073/pnas.1421580112 PMC4522803 26170307

[92] Liu L Inouye KE Allman WR Coleman AS Siddiqui S Hotamisligil GS. TACI-deficient macrophages protect mice against metaflammation and obesity-induced dysregulation of glucose homeostasis. Diabetes (2018) 67:1589–603. doi: 10.2337/db17-1089 PMC6054430 29871859

[93] Avery DT Kalled SL Ellyard JI Ambrose C Bixler SA Thien M. BAFF selectively enhances the survival of plasmablasts generated from human memory b cells. J Clin Invest (2003) 112:286–97. doi: 10.1172/JCI18025 PMC164292 12865416

[94] Novak AJ Darce JR Arendt BK Harder B Henderson K Kindsvogel W. Expression of BCMA, TACI, and BAFF-r in multiple myeloma: a mechanism for growth and survival. Blood (2004) 103:689–94. doi: 10.1182/blood-2003-06-2043 14512299

[95] Novak AJ Grote DM Stenson M Ziesmer SC Witzig TE Habermann TM. Expression of BLyS and its receptors in b-cell non-Hodgkin lymphoma: correlation with disease activity and patient outcome. Blood (2004) 104:2247–53. doi: 10.1182/blood-2004-02-0762 15251985

[96] Miyake T Abe M Tokumoto Y Hirooka M Furukawa S Kumagi T. 'B cell-activating factor is associated with the histological severity of nonalcoholic fatty liver disease'. Hepatol Int (2013) 7:539–47. doi: 10.1007/s12072-012-9345-8 26201785

[97] Nishikawa H Enomoto H Iwata Y Kishino K Shimono Y Hasegawa K. B-cell activating factor belonging to the tumor necrosis factor family and interferon-gamma-Inducible protein-10 in autoimmune hepatitis. Med (Baltimore) (2016) 95:e3194. doi: 10.1097/MD.0000000000003194 PMC4998411 27015216

[98] Nakamura Y Abe M Kawasaki K Miyake T Watanabe T Yoshida O. Depletion of b cell-activating factor attenuates hepatic fat accumulation in a murine model of nonalcoholic fatty liver disease. Sci Rep (2019) 9:977. doi: 10.1038/s41598-018-37403-y 30700810 PMC6353938

[99] Kaneko T Amano H Kawano S Minowa K Ando S Watanabe T. Increased serum concentration of BAFF/APRIL and IgA2 subclass in patients with mixed connective tissue disease complicated by interstitial lung disease. Mod Rheumatol (2014) 24:310–5. doi: 10.3109/14397595.2013.843748 24252051

[100] Francois A Gombault A Villeret B Alsaleh G Fanny M Gasse P. B cell activating factor is central to bleomycin- and IL-17-mediated experimental pulmonary fibrosis. J Autoimmun (2015) 56:1–11. doi: 10.1016/j.jaut.2014.08.003 25441030

[101] Matsushita T Fujimoto M Echigo T Matsushita Y Shimada Y Hasegawa M. Elevated serum levels of APRIL, but not BAFF, in patients with atopic dermatitis. Exp Dermatol (2008) 17:197–202. doi: 10.1111/j.1600-0625.2007.00642.x 17979975

[102] Ibrahim ZA Ghaly NR El-Tatawy RA Khalil SM El-Batch MM. A proliferation-inducing ligand in atopic dermatitis and vitiligo. Int J Dermatol (2014) 53:1073–9. doi: 10.1111/ijd.12176 24372078

[103] Mohamed Ezzat MH Mohammed AA Ismail RI Shaheen KY. 'High serum APRIL levels strongly correlate with disease severity in pediatric atopic eczema'. Int J Dermatol (2016) 55:e494–500. doi: 10.1111/ijd.13230 27061429

[104] Matsushita T Hasegawa M Yanaba K Kodera M Takehara K Sato S. Elevated serum BAFF levels in patients with systemic sclerosis: enhanced BAFF signaling in systemic sclerosis b lymphocytes. Arthritis Rheum (2006) 54:192–201. doi: 10.1002/art.21526 16385515

[105] Matsushita T Fujimoto M Hasegawa M Matsushita Y Komura K Ogawa F. BAFF antagonist attenuates the development of skin fibrosis in tight-skin mice. J Invest Dermatol (2007) 127:2772–80. doi: 10.1038/sj.jid.5700919 17581616

[106] Matsushita T Fujimoto M Hasegawa M Tanaka C Kumada S Ogawa F. Elevated serum APRIL levels in patients with systemic sclerosis: distinct profiles of systemic sclerosis categorized by APRIL and BAFF. J Rheumatol (2007) 34:2056–62. 17896803

[107] Bielecki M Kowal K Lapinska A Bernatowicz P Chyczewski L Kowal-Bielecka O. Increased production of a proliferation-inducing ligand (APRIL) by peripheral blood mononuclear cells is associated with antitopoisomerase I antibody and more severe disease in systemic sclerosis. J Rheumatol (2010) 37:2286–9. doi: 10.3899/jrheum.100454 20810514

[108] Francois A Chatelus E Wachsmann D Sibilia J Bahram S Alsaleh G. B lymphocytes and b-cell activating factor promote collagen and profibrotic markers expression by dermal fibroblasts in systemic sclerosis. Arthritis Res Ther (2013) 15:R168. doi: 10.1186/ar4352 24289101 PMC3978899

[109] Matsushita T Kobayashi T Mizumaki K Kano M Sawada T Tennichi M. 'BAFF inhibition attenuates fibrosis in scleroderma by modulating the regulatory and effector b cell balance'. Sci Adv (2018) 4:eaas9944. doi: 10.1126/sciadv.aas9944 30009261 PMC6040844

[110] Habibie H Adhyatmika A Schaafsma D Melgert BN. The role of osteoprotegerin (OPG) in fibrosis: its potential as a biomarker and/or biological target for the treatment of fibrotic diseases. Pharmacol Ther (2021) 228:107941. doi: 10.1016/j.pharmthera.2021.107941 34171336

[111] Fabrega E Orive A Garcia-Suarez C Garcia-Unzueta M Antonio Amado J Pons-Romero F. Osteoprotegerin and RANKL in alcoholic liver cirrhosis. Liver Int (2005) 25:305–10. doi: 10.1111/j.1478-3231.2005.01073.x 15780054

[112] Hao YL Bian ZL Ju LL Liu Y Qin G. RANK/RANKL acts as a protective factor by targeting cholangiocytes in primary biliary cholangitis. Dig Dis Sci (2020) 65:470–79. doi: 10.1007/s10620-019-05758-5 31377883

[113] Jin F Geng F Xu D Li Y Li T Yang X. Ac-SDKP attenuates activation of lung macrophages and bone osteoclasts in rats exposed to silica by inhibition of TLR4 and RANKL signaling pathways. J Inflammation Res (2021) 14:1647–60. doi: 10.2147/JIR.S306883 PMC8088302 33948088

[114] Delion M Braux J Jourdain ML Guillaume C Bour C Gangloff S. Overexpression of RANKL in osteoblasts: a possible mechanism of susceptibility to bone disease in cystic fibrosis. J Pathol (2016) 240:50–60. doi: 10.1002/path.4753 27235726

[115] Hao Y Tsuruda T Sekita-Hatakeyama Y Kurogi S Kubo K Sakamoto S. Cardiac hypertrophy is exacerbated in aged mice lacking the osteoprotegerin gene. Cardiovasc Res (2016) 110:62–72. doi: 10.1093/cvr/cvw025 26825553

[116] Giannandrea M Parks WC. Diverse functions of matrix metalloproteinases during fibrosis. Dis Model Mech (2014) 7:193–203. doi: 10.1242/dmm.012062 24713275 PMC3917240

[117] Tsuruda T Sekita-Hatakeyama Y Hao Y Sakamoto S Kurogi S Nakamura M. Angiotensin II stimulation of cardiac hypertrophy and functional decompensation in osteoprotegerin-deficient mice. Hypertension (2016) 67:848–56. doi: 10.1161/HYPERTENSIONAHA.115.06689 27001297

[118] Zhao H Yang H Geng C Chen Y Pang J Shu T. Role of IgE-FcepsilonR1 in pathological cardiac remodeling and dysfunction. Circulation (2021) 143:1014–30. doi: 10.1161/CIRCULATIONAHA.120.047852 33305586

[119] Loudon BL Ntatsaki E Newsome S Halliday B Lota A Ali A. Osteoprotegerin and myocardial fibrosis in patients with aortic stenosis. Sci Rep (2018) 8:14550. doi: 10.1038/s41598-018-32738-y 30266917 PMC6162228

[120] Kamimura D Suzuki T Furniss AL Griswold ME Kullo IJ Lindsey ML. Elevated serum osteoprotegerin is associated with increased left ventricular mass index and myocardial stiffness. J Cardiovasc Med (Hagerstown) (2017) 18:954–61. doi: 10.2459/JCM.0000000000000549 PMC6100745 28787318

[121] Wang Y Michiels T Setroikromo R van Merkerk R Cool RH Quax WJ. Creation of RANKL mutants with low affinity for decoy receptor OPG and their potential anti-fibrosis activity. FEBS J (2019) 286:3582–93. doi: 10.1111/febs.14925 PMC6852375 31081236

[122] McHugh RS Whitters MJ Piccirillo CA Young DA Shevach EM Collins M. 'CD4(+)CD25(+) immunoregulatory T cells: gene expression analysis reveals a functional role for the glucocorticoid-induced TNF receptor'. Immunity (2002) 16:311–23. doi: 10.1016/S1074-7613(02)00280-7 11869690

[123] Nocentini G Giunchi L Ronchetti S Krausz LT Bartoli A Moraca R. A new member of the tumor necrosis factor/nerve growth factor receptor family inhibits T cell receptor-induced apoptosis. Proc Natl Acad Sci U S A (1997) 94:6216–21. doi: 10.1073/pnas.94.12.6216 PMC21029 9177197

[124] Ronchetti S Zollo O Bruscoli S Agostini M Bianchini R Nocentini G. 'GITR, a member of the TNF receptor superfamily, is costimulatory to mouse T lymphocyte subpopulations'. Eur J Immunol (2004) 34:613–22. doi: 10.1002/eji.200324804 14991590

[125] Nocentini G Riccardi C. GITR: a modulator of immune response and inflammation. Adv Exp Med Biol (2009) 647:156–73. doi: 10.1007/978-0-387-89520-8_11 19760073

[126] Tone M Tone Y Adams E Yates SF Frewin MR Cobbold SP. Mouse glucocorticoid-induced tumor necrosis factor receptor ligand is costimulatory for T cells. Proc Natl Acad Sci U.S.A. (2003) 100:15059–64. doi: 10.1073/pnas.2334901100 PMC299905 14608036

[127] Shevach EM Stephens GL. The GITR-GITRL interaction: co-stimulation or contrasuppression of regulatory activity? Nat Rev Immunol (2006) 6:613–8. doi: 10.1038/nri1867 16868552

[128] Gravestein LA Nieland JD Kruisbeek AM Borst J. Novel mAbs reveal potent co-stimulatory activity of murine CD27. Int Immunol (1995) 7:551–7. doi: 10.1093/intimm/7.4.551 7547681

[129] Hurtado JC Kim YJ Kwon BS. Signals through 4-1BB are costimulatory to previously activated splenic T cells and inhibit activation-induced cell death. J Immunol (1997) 158:2600–9. doi: 10.4049/jimmunol.158.6.2600 9058792

[130] Gramaglia I Weinberg AD Lemon M Croft M. Ox-40 ligand: a potent costimulatory molecule for sustaining primary CD4 T cell responses. J Immunol (1998) 161:6510–7. doi: 10.4049/jimmunol.161.12.6510 9862675

[131] Kanamaru F Youngnak P Hashiguchi M Nishioka T Takahashi T Sakaguchi S. Costimulation via glucocorticoid-induced TNF receptor in both conventional and CD25+ regulatory CD4+ T cells. J Immunol (2004) 172:7306–14. doi: 10.4049/jimmunol.172.12.7306 15187106

[132] Shimizu J Yamazaki S Takahashi T Ishida Y Sakaguchi S. Stimulation of CD25(+)CD4(+) regulatory T cells through GITR breaks immunological self-tolerance. Nat Immunol (2002) 3:135–42. doi: 10.1038/ni759 11812990

[133] Kim SH Youn J. Rheumatoid fibroblast-like synoviocytes downregulate Foxp3 expression by regulatory T cells Via GITRL/GITR interaction. Immune Netw (2012) 12:217–21. doi: 10.4110/in.2012.12.5.217 PMC3509167 23213316

[134] Zhou X Bailey-Bucktrout SL Jeker LT Penaranda C Martinez-Llordella M Ashby M. Instability of the transcription factor Foxp3 leads to the generation of pathogenic memory T cells *in vivo* . Nat Immunol (2009) 10:1000–7. doi: 10.1038/ni.1774 PMC2729804 19633673

[135] Chen X Wu Y Wang Y Chen L Zheng W Zhou S. Human menstrual blood-derived stem cells mitigate bleomycin-induced pulmonary fibrosis through anti-apoptosis and anti-inflammatory effects. Stem Cell Res Ther (2020) 11:477. doi: 10.1186/s13287-020-01926-x 33176882 PMC7656201

[136] Rueschenbaum S Ciesek S Queck A Widera M Schwarzkopf K Brune B. Dysregulated adaptive immunity is an early event in liver cirrhosis preceding acute-on-Chronic liver failure. Front Immunol (2020) 11:534731. doi: 10.3389/fimmu.2020.534731 33574809 PMC7870861

[137] Cuzzocrea S Ronchetti S Genovese T Mazzon E Agostini M Di Paola R. Genetic and pharmacological inhibition of GITR-GITRL interaction reduces chronic lung injury induced by bleomycin instillation. FASEB J (2007) 21:117–29. doi: 10.1096/fj.06-6611com 17135359

[138] Motta AC Vissers JL Gras R Van Esch BC Van Oosterhout AJ Nawijn MC. 'GITR signaling potentiates airway hyperresponsiveness by enhancing Th2 cell activity in a mouse model of asthma'. Respir Res (2009) 10:93. doi: 10.1186/1465-9921-10-93 19811658 PMC2767348

[139] Prasse A Pechkovsky DV Toews GB Jungraithmayr W Kollert F Goldmann T. A vicious circle of alveolar macrophages and fibroblasts perpetuates pulmonary fibrosis via CCL18. Am J Respir Crit Care Med (2006) 173:781–92. doi: 10.1164/rccm.200509-1518OC 16415274

[140] Paterson DJ Jefferies WA Green JR Brandon MR Corthesy P Puklavec M. Antigens of activated rat T lymphocytes including a molecule of 50,000 Mr detected only on CD4 positive T blasts. Mol Immunol (1987) 24:1281–90. doi: 10.1016/0161-5890(87)90122-2 2828930

[141] Stuber E Neurath M Calderhead D Fell HP Strober W. Cross-linking of OX40 ligand, a member of the TNF/NGF cytokine family, induces proliferation and differentiation in murine splenic b cells. Immunity (1995) 2:507–21. doi: 10.1016/1074-7613(95)90031-4 7749983

[142] Ohshima Y Tanaka Y Tozawa H Takahashi Y Maliszewski C Delespesse G. Expression and function of OX40 ligand on human dendritic cells. J Immunol (1997) 159:3838–48. doi: 10.4049/jimmunol.159.8.3838 9378971

[143] Akiba H Oshima H Takeda K Atsuta M Nakano H Nakajima A. CD28-independent costimulation of T cells by OX40 ligand and CD70 on activated b cells. J Immunol (1999) 162:7058–66. doi: 10.4049/jimmunol.162.12.7058 10358148

[144] Rogers PR Song J Gramaglia I Killeen N Croft M. OX40 promotes bcl-xL and bcl-2 expression and is essential for long-term survival of CD4 T cells. Immunity (2001) 15:445–55. doi: 10.1016/S1074-7613(01)00191-1 11567634

[145] Cunninghame Graham DS Graham RR Manku H Wong AK Whittaker JC Gaffney PM. Polymorphism at the TNF superfamily gene TNFSF4 confers susceptibility to systemic lupus erythematosus. Nat Genet (2008) 40:83–9. doi: 10.1038/ng.2007.47 PMC3705866 18059267

[146] Gourh P Arnett FC Tan FK Assassi S Divecha D Paz G. Association of TNFSF4 (OX40L) polymorphisms with susceptibility to systemic sclerosis. Ann Rheum Dis (2010) 69:550–5. doi: 10.1136/ard.2009.116434 PMC2927683 19778912

[147] Rabieyousefi M Soroosh P Satoh K Date F Ishii N Yamashita M. Indispensable roles of OX40L-derived signal and epistatic genetic effect in immune-mediated pathogenesis of spontaneous pulmonary hypertension. BMC Immunol (2011) 12:67. doi: 10.1186/1471-2172-12-67 22171643 PMC3269997

[148] Elhai M Avouac J Hoffmann-Vold AM Ruzehaji N Amiar O Ruiz B. OX40L blockade protects against inflammation-driven fibrosis. Proc Natl Acad Sci U.S.A. (2016) 113:E3901–10. doi: 10.1073/pnas.1523512113 PMC4941508 27298374

[149] Foks AC van Puijvelde GH Bot I ter Borg MN Habets KL Johnson JL. Interruption of the OX40-OX40 ligand pathway in LDL receptor-deficient mice causes regression of atherosclerosis. J Immunol (2013) 191:4573–80. doi: 10.4049/jimmunol.1200708 24068673

[150] Hutton AJ Polak ME Spalluto CM Wallington JC Pickard C Staples KJ. Human lung fibroblasts present bacterial antigens to autologous lung Th cells. J Immunol (2017) 198:110–18. doi: 10.4049/jimmunol.1600602 27895174

[151] Han BK Olsen NJ Bottaro A. The CD27-CD70 pathway and pathogenesis of autoimmune disease. Semin Arthritis Rheum (2016) 45:496–501. doi: 10.1016/j.semarthrit.2015.08.001 26359318

[152] Lens SM Tesselaar K van Oers MH van Lier RA. Control of lymphocyte function through CD27-CD70 interactions. Semin Immunol (1998) 10:491–9. doi: 10.1006/smim.1998.0154 9826582

[153] Agematsu K Hokibara S Nagumo H Komiyama A. CD27: a memory b-cell marker. Immunol Today (2000) 21:204–6. doi: 10.1016/S0167-5699(00)01605-4 10782048

[154] Hintzen RQ de Jong R Hack CE Chamuleau M de Vries EF ten Berge IJ. A soluble form of the human T cell differentiation antigen CD27 is released after triggering of the TCR/CD3 complex. J Immunol (1991) 147:29–35. doi: 10.4049/jimmunol.147.1.29 1646845

[155] Loenen WA De Vries E Gravestein LA Hintzen RQ Van Lier RA Borst J. The CD27 membrane receptor, a lymphocyte-specific member of the nerve growth factor receptor family, gives rise to a soluble form by protein processing that does not involve receptor endocytosis. Eur J Immunol (1992) 22:447–55. doi: 10.1002/eji.1830220224 1311261

[156] Kato K Chu P Takahashi S Hamada H Kipps TJ. Metalloprotease inhibitors block release of soluble CD27 and enhance the immune stimulatory activity of chronic lymphocytic leukemia cells. Exp Hematol (2007) 35:434–42. doi: 10.1016/j.exphem.2006.10.018 17309824

[157] Sugita K Hirose T Rothstein DM Donahue C Schlossman SF Morimoto C. CD27, a member of the nerve growth factor receptor family, is preferentially expressed on CD45RA+ CD4 T cell clones and involved in distinct immunoregulatory functions. J Immunol (1992) 149:3208–16. doi: 10.4049/jimmunol.149.10.3208 1358967

[158] Xiao Y Hendriks J Langerak P Jacobs H Borst J. CD27 is acquired by primed b cells at the centroblast stage and promotes germinal center formation. J Immunol (2004) 172:7432–41. doi: 10.4049/jimmunol.172.12.7432 15187121

[159] Jacquot S Kobata T Iwata S Morimoto C Schlossman SF. CD154/CD40 and CD70/CD27 interactions have different and sequential functions in T cell-dependent b cell responses: enhancement of plasma cell differentiation by CD27 signaling. J Immunol (1997) 159:2652–7. doi: 10.4049/jimmunol.159.6.2652 9300684

[160] Agematsu K Nagumo H Oguchi Y Nakazawa T Fukushima K Yasui K. Generation of plasma cells from peripheral blood memory b cells: synergistic effect of interleukin-10 and CD27/CD70 interaction. Blood (1998) 91:173–80. doi: 10.1182/blood.V91.1.173 9414282

[161] Oshikawa Y Makino T Nakayama M Sawamura S Makino K Kajihara I. Increased CD27 expression in the skins and sera of patients with systemic sclerosis. Intractable Rare Dis Res (2020) 9:99–103. doi: 10.5582/irdr.2020.03017 32494557 PMC7263984

[162] Tran-Nguyen TK Xue J Feghali-Bostwick C Sciurba FC Kass DJ Duncan SR. CD70 activation decreases pulmonary fibroblast production of extracellular matrix proteins. Am J Respir Cell Mol Biol (2020) 63:255–65. doi: 10.1165/rcmb.2019-0450OC PMC7397763 32320626

[163] Heller F Lindenmeyer MT Cohen CD Brandt U Draganovici D Fischereder M. The contribution of b cells to renal interstitial inflammation. Am J Pathol (2007) 170:457–68. doi: 10.2353/ajpath.2007.060554 PMC1851872 17255314

[164] Doi H Iyer TK Carpenter E Li H Chang KM Vonderheide RH. Dysfunctional b-cell activation in cirrhosis resulting from hepatitis c infection associated with disappearance of CD27-positive b-cell population. Hepatology (2012) 55:709–19. doi: 10.1002/hep.24689 PMC3245804 21932384

[165] Chang LY Li Y Kaplan DE. Endotoxemia contributes to CD27+ memory b-cell apoptosis via enhanced sensitivity to fas ligation in patients with cirrhosis. Sci Rep (2016) 6:36862. doi: 10.1038/srep36862 27857173 PMC5114671

[166] Ong S Ligons DL Barin JG Wu L Talor MV Diny N. Natural killer cells limit cardiac inflammation and fibrosis by halting eosinophil infiltration. Am J Pathol (2015) 185:847–61. doi: 10.1016/j.ajpath.2014.11.023 PMC4348473 25622543

[167] Luo Y Wang Y Shu Y Lu Q Xiao R. Epigenetic mechanisms: an emerging role in pathogenesis and its therapeutic potential in systemic sclerosis. Int J Biochem Cell Biol (2015) 67:92–100. doi: 10.1016/j.biocel.2015.05.023 26043891

[168] Wiley SR Schooley K Smolak PJ Din WS Huang CP Nicholl JK. Identification and characterization of a new member of the TNF family that induces apoptosis. Immunity (1995) 3:673–82. doi: 10.1016/1074-7613(95)90057-8 8777713

[169] Zamai L Ahmad M Bennett IM Azzoni L Alnemri ES Perussia B. Natural killer (NK) cell-mediated cytotoxicity: differential use of TRAIL and fas ligand by immature and mature primary human NK cells. J Exp Med (1998) 188:2375–80. doi: 10.1084/jem.188.12.2375 PMC2212426 9858524

[170] Griffith TS Wiley SR Kubin MZ Sedger LM Maliszewski CR Fanger NA. Monocyte-mediated tumoricidal activity via the tumor necrosis factor-related cytokine, TRAIL. J Exp Med (1999) 189:1343–54. doi: 10.1084/jem.189.8.1343 PMC2193036 10209050

[171] Arbour N Rastikerdar E McCrea E Lapierre Y Dorr J Bar-Or A. Upregulation of TRAIL expression on human T lymphocytes by interferon beta and glatiramer acetate. Mult Scler (2005) 11:652–7. doi: 10.1191/1352458505ms1222oa 16320724

[172] Pan G O'Rourke K Chinnaiyan AM Gentz R Ebner R Ni J. The receptor for the cytotoxic ligand TRAIL. Science (1997) 276:111–3. doi: 10.1126/science.276.5309.111 9082980

[173] MacFarlane M Ahmad M Srinivasula SM Fernandes-Alnemri T Cohen GM Alnemri ES. Identification and molecular cloning of two novel receptors for the cytotoxic ligand TRAIL. J Biol Chem (1997) 272:25417–20. doi: 10.1074/jbc.272.41.25417 9325248

[174] Suliman A Lam A Datta R Srivastava RK. Intracellular mechanisms of TRAIL: apoptosis through mitochondrial-dependent and -independent pathways. Oncogene (2001) 20:2122–33. doi: 10.1038/sj.onc.1204282 11360196

[175] Pan G Ni J Wei YF Yu G Gentz R Dixit VM. An antagonist decoy receptor and a death domain-containing receptor for TRAIL. Science (1997) 277:815–8. doi: 10.1126/science.277.5327.815 9242610

[176] Marsters SA Sheridan JP Pitti RM Huang A Skubatch M Baldwin D. A novel receptor for Apo2L/TRAIL contains a truncated death domain. Curr Biol (1997) 7:1003–6. doi: 10.1016/S0960-9822(06)00422-2 9382840

[177] Emery JG McDonnell P Burke MB Deen KC Lyn S Silverman C. Osteoprotegerin is a receptor for the cytotoxic ligand TRAIL. J Biol Chem (1998) 273:14363–7. doi: 10.1074/jbc.273.23.14363 9603945

[178] Schneider P Olson D Tardivel A Browning B Lugovskoy A Gong D. Identification of a new murine tumor necrosis factor receptor locus that contains two novel murine receptors for tumor necrosis factor-related apoptosis-inducing ligand (TRAIL). J Biol Chem (2003) 278:5444–54. doi: 10.1074/jbc.M210783200 12466268

[179] Ashkenazi A Pai RC Fong S Leung S Lawrence DA Marsters SA. Safety and antitumor activity of recombinant soluble Apo2 ligand. J Clin Invest (1999) 104:155–62. doi: 10.1172/JCI6926 PMC408479 10411544

[180] Walczak H Miller RE Ariail K Gliniak B Griffith TS Kubin M. Tumoricidal activity of tumor necrosis factor-related apoptosis-inducing ligand *in vivo* . Nat Med (1999) 5:157–63. doi: 10.1038/5517 9930862

[181] Oh Y Park O Swierczewska M Hamilton JP Park JS Kim TH. Systemic PEGylated TRAIL treatment ameliorates liver cirrhosis in rats by eliminating activated hepatic stellate cells. Hepatology (2016) 64:209–23. doi: 10.1002/hep.28432 PMC4917440 26710118

[182] Novo E Marra F Zamara E Valfre di Bonzo L Monitillo L Cannito S. Overexpression of bcl-2 by activated human hepatic stellate cells: resistance to apoptosis as a mechanism of progressive hepatic fibrogenesis in humans. Gut (2006) 55:1174–82. doi: 10.1136/gut.2005.082701 PMC1856252 16423888

[183] Park JS Oh Y Park YJ Park O Yang H Slania S. Targeting of dermal myofibroblasts through death receptor 5 arrests fibrosis in mouse models of scleroderma. Nat Commun (2019) 10:1128. doi: 10.1038/s41467-019-09101-4 30850660 PMC6408468

[184] Collison AM Sokulsky LA Sherrill JD Nightingale S Hatchwell L Talley NJ. TNF-related apoptosis-inducing ligand (TRAIL) regulates midline-1, thymic stromal lymphopoietin, inflammation, and remodeling in experimental eosinophilic esophagitis. J Allergy Clin Immunol (2015) 136:971–82. doi: 10.1016/j.jaci.2015.03.031 PMC4600423 25981737

